# Megathrust locking encoded in subduction landscapes

**DOI:** 10.1126/sciadv.adl4286

**Published:** 2024-04-26

**Authors:** Bar Oryan, Jean-Arthur Olive, Romain Jolivet, Luca C. Malatesta, Boris Gailleton, Lucile Bruhat

**Affiliations:** ^1^ Laboratoire de Géologie, École Normale Supérieure– PSL, CNRS UMR 8538, Paris, France.; ^2^ Scripps Institution of Oceanography, UC San Diego, La Jolla, CA 92093, USA.; ^3^ Institut Universitaire de France, 1 rue Descartes, 75006 Paris, France.; ^4^ Earth Surface Process Modelling, GFZ German Research Center for Geosciences, 14473 Potsdam, Germany.; ^5^ Université de Rennes, Géosciences Rennes, UMR 6118, 35000 Rennes, France.; ^6^ Group Risk Management, AXA, Paris, France.

## Abstract

Locked areas of subduction megathrusts are increasingly found to coincide with landscape features sculpted over hundreds of thousand years, yet the mechanisms that underlie such correlations remain elusive. We show that interseismic locking gradients induce increments of irreversible strain across the overriding plate manifested predominantly as distributed seismicity. Summing these increments over hundreds of earthquake cycles produces a spatially variable field of uplift representing the unbalance of co-, post-, and interseismic strain. This long-term uplift explains first-order geomorphological features of subduction zones such as the position of the continental erosive shelf break, the distribution of marine terraces and peninsulas, and the profile of forearc rivers. Inelastic yielding of the forearc thus encodes short-term locking patterns in subduction landscapes, hinting that megathrust locking is stable over multiple earthquake cycles and highlighting the role geomorphology can play in constraining Earth’s greatest source of seismic hazard.

## Teaser

Interseismic locking on megathrusts permanently strains the overriding plate, leaving a distinct landscape footprint.

## INTRODUCTION

**Fig. 1.**
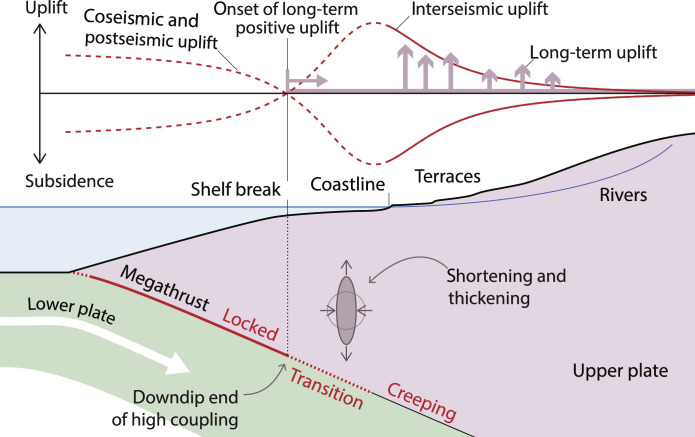
Signatures of the short-term megathrust cycle in long-term forearc morphology. Elastic surface displacement during the interseismic, postseismic, and coseismic periods is denoted by red curves. Evidence for permanent surface deformation recorded by rivers, terraces, and shelf breaks is marked by pink arrows.

The largest earthquakes on Earth occur at subduction zones, where a dense tectonic plate sinks into the mantle, sliding below another plate (*1*, *2*). The plate interface, or megathrust (Fig. 1), is populated by asperities where the two plates intermittently stick together for tens to hundreds of years, until they break and generate a megathrust rupture (*1*). Gradual interseismic loading typically produces slow surface uplift landward of the locked asperities, followed by rapid co-and postseismic motion that mirrors interseismic displacements (*3*). This pattern presumably repeats itself over hundreds of thousands of years as the upper plate experiences countless cycles of loading and unloading. To mitigate the hazard associated with megathrust earthquakes, geodesists routinely measure rates of interseismic surface displacement and invert them for a distribution of slip deficit with respect to the convergence rate along the subduction interface (*4*–*6*). This helps locate locked asperities, also known as highly coupled regions, and evaluate the seismic risk they pose. This approach is inherently limited by short temporal span and uneven spatial coverage of geodetic data (*7*, *8*). Specifically, it is unclear whether the spatial pattern of megathrust locking persists or evolves over multiple seismic cycles. Knowing this would provide valuable insight into the physical mechanisms that underlie megathrust locking (*9*).

### Megathrust locking leaves distinct geomorphological footprints

**Fig. 2.**
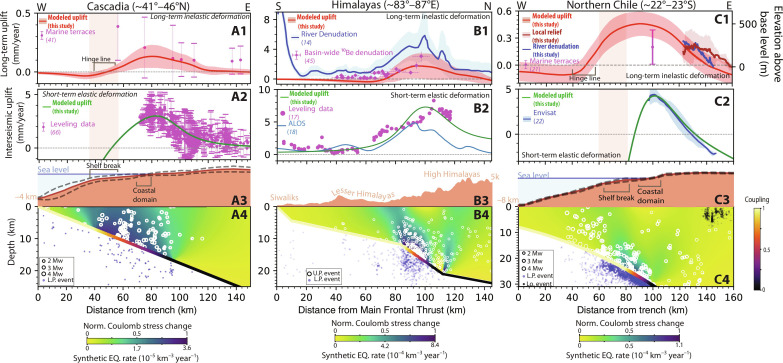
Short- and long-term uplift in three studied subduction zones. (**A**), (**B**), and (**C**) show results for Cascadia, the Himalayas, and northern Chile, respectively. (A1 to C1) Long-term uplift computed by our model and recorded by marine terraces (*21*, *41*), basin-wide denudation rates (*45*), and rivers (*14*). (A2 to C2) Interseismic uplift inferred by our models and documented by leveling data (*17*, *92*) and InSAR (*18*, *22*). Continuous curves overlapped by light filling denote dataset mean and SD, respectively (texts S3 to S5). Error bars mark 1 SD. Light brown background marks the position of the shelf break in the swath. (A3 to C3) Mean topography of the transect where earthquakes are recorded. Dashed lines denote 1 SD (figs. S3, S5, and S6 and texts S3 and S4). (A4 to C4) Normalized Coulomb stress change and rate of synthetic earthquakes used to compute inelastic uplift. Recorded seismicity is marked by circles (*33*, *42*–*44*). Subduction zone interfaces are color coded by the coupling model we used. For a full description of model parameters, see table S1. U.P., upper plate; L.P, lower plate; Lo, local event.

Geomorphological observations, on the other hand, cover timescales of hundreds of thousands of years that are longer than seismic cycles (hundreds of years) but shorter than the millions of years over which the geological architecture of subduction margins evolves (*10*). A growing body of work suggests that the spatial pattern of megathrust locking between large earthquakes leaves a distinct footprint in subduction landscapes. For example, locked patches associated with great subduction zone earthquakes are typically overlain by forearc basins (*11*) and associated with negative topography (or gravity) anomalies (*12*), suggesting that regions experiencing interseismic subsidence also undergo long-term subsidence over many cycles (Fig. 1). Closer to the land, the seaward end of the erosive continental shelf (shelf break) commonly overlies the downdip end of fully locked megathrust regions (*13*) (Figs. 1; 2, A1 and A3; and 3B). The shelf break can be regarded as a hinge line that marks the beginning of a landward domain experiencing sustained rock uplift, where new rocks are continually raised to shallow levels and undergo wave erosion. This pattern of long-term vertical displacement bears similarities with that observed during the interseismic phase of the megathrust cycle (Fig. 1). This resemblance is particularly notable in the Himalayan subduction zone, where the field of rock uplift that has prevailed over the past hundreds of thousand years (kyr) can be inferred from fluvial incision rates (*14*), river profiles (*15*), or changes in valley width (*16*). This field features a broad peak of rapid rock uplift above the downdip end of full megathrust locking (Fig. 2B1). A similar peak exists in the shape of ongoing, interseismic vertical displacements measured over decades (*17*, *18*) (Fig. 2B2). Intriguing correlations have also been reported between along-strike changes in subduction morphology and present-day interseismic deformation. In Central and South American subduction zones, the position of peninsulas, for example, coincides with regions of reduced megathrust locking (*19*, *20*). Furthermore, Quaternary uplift rates along the Chilean coast recorded by marine terraces (*21*) systematically amount to 4 to 8% of present-day interseismic uplift rates (*22*). Last, areas of faster Quaternary uplift are also associated with greater upper-plate seismic activity during the interseismic phase (*23*). These observations hint at a close link between the processes fueling megathrust earthquakes over timescales of decades to centuries and those shaping subduction landscapes over hundreds of thousands of years.

**Fig. 3.**
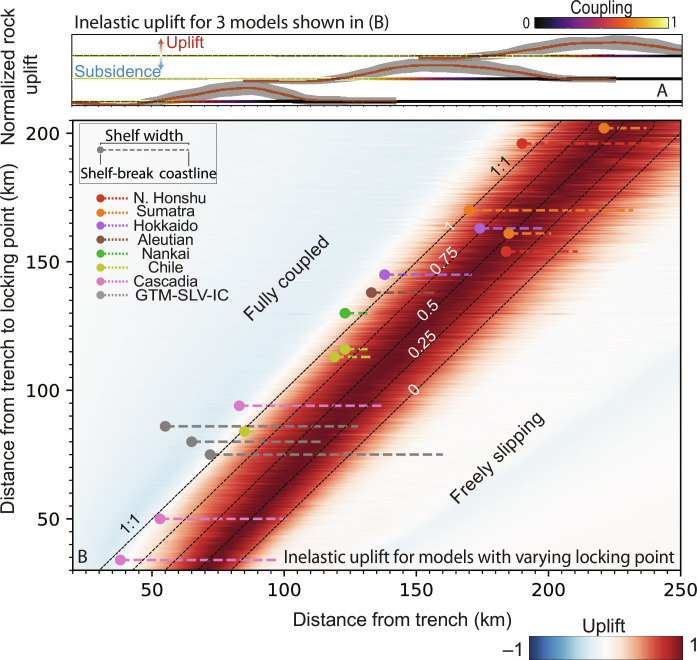
Global correlation between our predicted field of uplift, coupling distribution, and shelf break. (**B**) Mean inelastic surface uplift for 760 models with varying extents of fully locked zones, spanning a range of 30 to 205 km from the trench. Each row along the *y* axis shows the mean inelastic uplift of a model (color). The documented positions of the shelf-break and coastline in a number of forearcs are marked by circles and adjacent lines, respectively (*13*). The mean and SD of uplift are displayed in (**A**) for three different models. The coupling profile used to compute these three models is shown at the base of each of the uplift patterns. For a complete description of model parameters, see table S1.

These connections between short- and long-term timescales are often interpreted as manifestations of unbalanced earthquake cycles (*15*, *24*–*26*), meaning that interseismic and co/postseismic displacements do not cancel each other but sum into a poorly known field of residual interseismic uplift/subsidence that shapes the forearc landscape (*13*, *15*, *20*, *27*). This interpretation is, however, at odds with the widely used backslip model (*5*), which is the standard model for characterizing deformation associated with earthquake cycles (*8*, *22*, *28*, *29*). This framework assumes that off-fault deformation is purely elastic, implying that aside from slip on the megathrust, no permanent strain—and consequently, no long-term rock uplift—is expected. We instead propose that increments of nonrecoverable, distributed brittle deformation in the upper plate accumulate during the interseismic phase of the megathrust across subduction forearcs, in a manner that is strongly modulated by megathrust locking, and account for most of the geomorphological observations described above.

## RESULTS

### From interseismic locking to long-term uplift

Inelastic deformation mechanisms in the brittle forearc are activated when stresses reach specific thresholds during the interseismic phase (*30*). It was previously noted (*31*) that down-dip gradients in the degree of megathrust locking are a straightforward way of generating stress concentrations in the upper plate. Furthermore, previous work (*23*, *27*) postulated a link between long-term uplift and upper-plate seismicity. We propose a model integrating these ideas into a workflow that relates short-term locking state to long-term uplift (see Materials and Methods). We illustrate it below through the example of the Cascadia, Chile, and the Himalayan subduction zones.

### Summing locking-driven seismicity to explain long-term forearc surface motion

The current state of locking on a megathrust (Fig. 2A4) can be inferred by inverting geodetically determined surface displacements within the backslip framework (Fig. 2A2). We use this model to determine the Coulomb stress change imparted by the locking distribution on the forearc wedge, assumed rheologically homogeneous (Fig. 2A4). We assume the upper plate is near a state of overall compressive yielding (*32*) allowing us to disregard Coulomb stress changes that drive extensional slip such as during the coseismic and postseismic periods. Consequently, we exclusively focus on the Coulomb stress change prompting reverse fault slip, which builds up during interseismic period. The largest compressive stress rates occur above the transition zone (Fig. 2, A4, B4, and C4, and fig. S1) connecting the fully locked and fully slipping portions of the megathrust. This is also the area where seismicity tends to cluster, for instance in the Cascadia forearc (white circles in Fig. 2A4), as revealed by a recent 4-year ocean bottom seismometers (OBS) survey (*33*). We hypothesize that this seismicity is a signature of the upper plate yielding between large megathrust earthquakes, which over longer timescales shortens and thickens the forearc in a coherent, nonreversible manner (Fig. 1). To quantify this deformation, we generate millions of synthetic earthquakes spanning thousands of years and dozens of seismic cycles. We spatially distribute these synthetic earthquakes within the forearc by assuming a linear relationship between Coulomb stress rates and seismicity rates (*34*, *35*) (see Materials and Methods). We assign these synthetic events a seismic moment randomly drawn from the locally measured Gutenberg-Richter distribution. Each event is then associated with a rectangular fault patch and a reverse-slip vector consistent with empirical moment-displacement scalings (*36*). Fault patches are assumed to have optimal landward or seaward dips with respect to a state of horizontal compression (i.e., dips of ~30°). By adding the elastic displacement fields caused by each individual earthquake (*37*, *38*), we effectively compute the cumulative surface motion resulting from seismicity over thousands of years representing numerous seismic cycles. We postulate that this distributed inelastic forearc deformation cannot be recovered when the megathrust slips and therefore constitutes a reasonable proxy for the long-term uplift field that shapes the landscape.

### Application to the Cascadia subduction zone

To model a two-dimensional (2D) cross section of the Cascadia subduction zone, we generate 1.9 million synthetic earthquakes distributed spatially according to slip deficit along the interface (*8*) and the Gutenberg distribution observed by a local seismic catalog (*33*) (Fig. 2A4). Given current seismicity rates in the region (figs. S3 and S4 and text S3), this synthetic catalog covers ~72,000 years, which amounts to ~140 earthquake cycles assuming ~500-year cycles (*39*). The displacement fields of individual forearc earthquakes sum coherently (*40*) into a broad peak of rapid surface uplift located above the locking transition zone (Fig. 2A1). A zone of subdued uplift flanks this peak landward, with a seaward region of moderate subsidence. We attribute this pattern to the clustering of thrust events in the region of highest Coulomb stress rates, effectively acting as a deep zone of horizontal shortening that lifts the surface and produces gentle downward motion in the far field. Our predicted field of long-term uplift produces a hinge line between seaward subsidence and landward uplift that coincides with the edge of the Cascadia shelf, and the downdip end of the fully locked zone (Fig. 2, A1 and A3), supporting previous interpretation (*13*). Furthermore, the uplift rates we infer are on the same order of magnitude as those recorded by marine terraces (*41*) at different distances from the trench (~0.1 mm/year; Fig. 2A1). We thus suggest that coherent stacking of displacements due to upper-plate seismicity is a viable mechanism to explain long-term deformation of the Cascadia forearc.

### Application to the Himalayan collision zone and Chilean subduction zone

We further test our model by applying it to the Himalayan and northern Chilean subduction zones (Fig. 2, B and C, and figs. S6 to S8) where datasets documenting slip-deficient distributions (*22*, *28*), upper-plate seismicity (*42*–*44*), interseismic displacements (*17*, *18*, *22*), and long-term rock uplift (*14*, *21*, *45*) are available. The Himalayan example (Fig. 2B) is in many ways similar to Cascadia, with upper-plate seismicity clustering where the locking distribution imparts the highest compressive stress rates, i.e., above the locking transition zone (Fig. 2B2). The long-term uplift field computed from 0.5 million synthetic events spanning 2000 years [10 ~200-year-long cycles; (*46*)] closely resembles that inferred from river incision rates (*14*), fluvial geometry (*15*), as well as catchment-wide erosion rates (*47*). Specifically, they all involve a broad peak above the locking transition zone at roughly 100 km north of the Main Frontal Thrust and long-term rates on the order of mm/year (Fig. 2B1). Our model, however, does not account for rapid rock uplift in the Siwaliks (Fig. 2B), which we attribute to the geometry of the Main Frontal Thrust (*48*) rather than to inelastic interseismic deformation within the upper plate.

In northern Chile, the locking transition zone directly underlies the coastal domain (*22*). Consequently, the surface displacements from 2.9 million synthetic events spanning 17 thousand years [68 cycles assuming ~250-year cycles (*49*)] stack into an uplift field with a broad peak centered on the coast (Fig. 2, C1 and C3), with coastal uplift rates of ~0.5 mm/year, slightly exceeding the rates inferred from marine terraces (*21*). We further predict a gradual, landward decrease in long-term uplift that is consistent with regional proxies for uplift derived from the topography of the coastal range and the pattern of river incision across it (see Materials and Methods; Fig. 2C1). However, the anticipated hinge line, marking the transition from seaward subsidence to coastal uplift, is located approximately 15 km landward of the continental shelf’s edge. We also note that clusters of forearc seismicity do not exclusively occur in areas where we predict high Coulomb stress rates (Fig. 2C4). Overall, the slight mismatch between our model and geomorphological data suggests that additional mechanisms beyond inelastic deformation induced by locking gradients contribute to the morphology of the Chilean forearc.

### Additional sources of complexity in forearc morphology

Slip on faults of all sizes distributed across the forearc and activated by locking-induced compression is hardly the only inelastic deformation mechanism that can sculpt forearc landscapes. Other possible ways of permanently straining the upper plate between large earthquakes include pressure solution (*50*) and brittle creep (*51*) taking place across an heterogeneous forearc (*52*). Nonrecoverable strain may also accrue during megathrust ruptures, in the form of shallow plastic yielding (*53*), shallow fracturing (*54*), broad outer wedge failure (*55*), or fracturing in the damage zone of rupturing asperities (*56*). Fold-and-thrust belts may also generate nonrecoverable deformation during the postseismic (*57*, *58*) and interseismic (*59*) periods. Whether these mechanisms would imprint a spatially coherent mark in subduction landscapes however remains unclear. It is also unclear how strain imbalance documented following a few megathrust events (*21*) such as the 2015 Gorkha earthquake (*46*) would affect the long-term strain buildup of the forearc. Furthermore, the ability of inelastic interseismic deformation to reproduce long-term coastal uplift may suggest that co- and postseismic processes are of a lesser importance for the cases we have studied. Processes not directly related to the seismic cycle such as underplating (*60*, *61*) could plausibly result in a local maximum in forearc uplift. Underplating may require the development of secondary fault systems above the megathrust that enable the aforementioned mass transfers (*60*). Stress changes caused by locking gradients could well influence the development of such structures and contribute to the link between seismic cycle deformation and long-term uplift.

Our model also has inherent limitations, which relate to a number of simplifying assumptions. Among them is the treatment of the upper plate as a uniform elastic half-space on the verge of compressional failure. In reality, the forearc may be away from compressional yield with entire regions experiencing horizontal deviatoric tension (*10*, *62*). Repeated failure may also damage and weaken the forearc in a highly heterogeneous fashion that cannot be simply accounted for in our model. Another shortcoming of our approach is that it does not self-consistently predict the absolute magnitude of uplift (only its dimensionless shape). An absolute rate requires knowledge of the Gutenberg-Richter A-value, i.e., the absolute seismicity rates for the region of interest. Improvements of our model would necessitate a rheology-based determination of yielding regions and inelastic strain rates, in a manner that is self-consistent with the stress rates imposed by locking.

### Broader implications for subduction landscapes

**Fig. 4.**
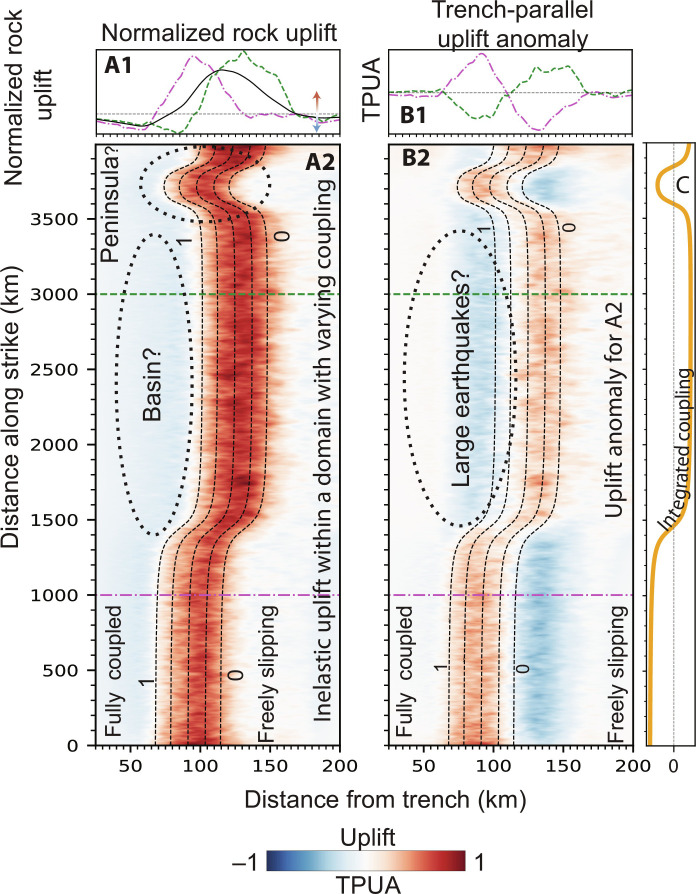
Global correlation between our predicted field of uplift, peninsulas, and uplift anomalies. (**A2**) Inelastic uplift along a 4000-km-long domain with varying coupling. (**A1**) Green and Magenta curves show the uplift along two lines shown in (B2). Black line shows the trench-parallel average uplift along the domain. (**B2**) Trench-parallel uplift anomaly. (**B1**) Green and magenta curves mark the Trench-parallel uplift anomaly along two lines shown in (B2). (**C**) Integrated coupling in the downdip direction for (A2) and (B2). Thin dashed lines mark the coupling used in computing uplift shown in (A2) and (B2). For a complete description of model parameters, see table S1.

In spite of its limitations, our model provides a first-order explanation for the common traits between long-term and interseismic uplift in Cascadia, the Himalayas, and northern Chile. To explore the wider implications of observed trends in subduction zones, we conduct 760 additional model runs. Rather than focus on a specific subduction context, these simulations are designed to probe the global relationship between slip deficit and long-term uplift. Each model is assigned a unique locking distribution, with the fully locked zone extending from 30 to 205 km from the trench. We examine the dimensionless shape of the long-term uplift field produced by each of the models (Fig. 3B). Consistent with our prior results (Fig. 2), the broad peak of long-term uplift systematically overlies the locking transition zone, regardless of its depth, and the hinge line between seaward subsidence and long-term uplift follows the downdip end of the fully locked zone. Our model thereby accounts for the global colocation of the downdip end of locking and shelf breaks (*13*) through the location of the high stress rate area and resulting inelastic strain (Fig. 3B). The relationship between areas with reduced slip deficit and the occurrence of peninsulas can be seen as a corollary to this phenomenon (*19*, *20*). To illustrate this, we compute the long-term uplift field within a 4000-km-long (along-trench) domain that includes a zone of anomalously low coupling, where the locking transition zone is closer to the trench (Fig. 4A2). There, the model produces an uplift peak that is closer to the trench and shifts the shelf break and the coast seaward, which could result in a peninsula (Fig. 4A). Conversely, an area prone to large seismic ruptures, i.e., with an extensive locked zone (and a locking transition zone further away from the trench), will tend to subside long term. Sustained subsidence (Fig. 4A2) over many seismic cycles may contribute to the formation of forearc basins (*11*). Last, we calculate uplift rate anomalies relative to the cross-trench uplift profile averaged along our entire domain. This yields negative uplift anomalies over regions where full locking extends further downdip of the trench (Fig. 4, B1 and B2), and may provide an explanation for the negative topography/gravity anomalies reported above the areas of large coseismic ruptures (*12*).

## DISCUSSION

**Fig. 5.**
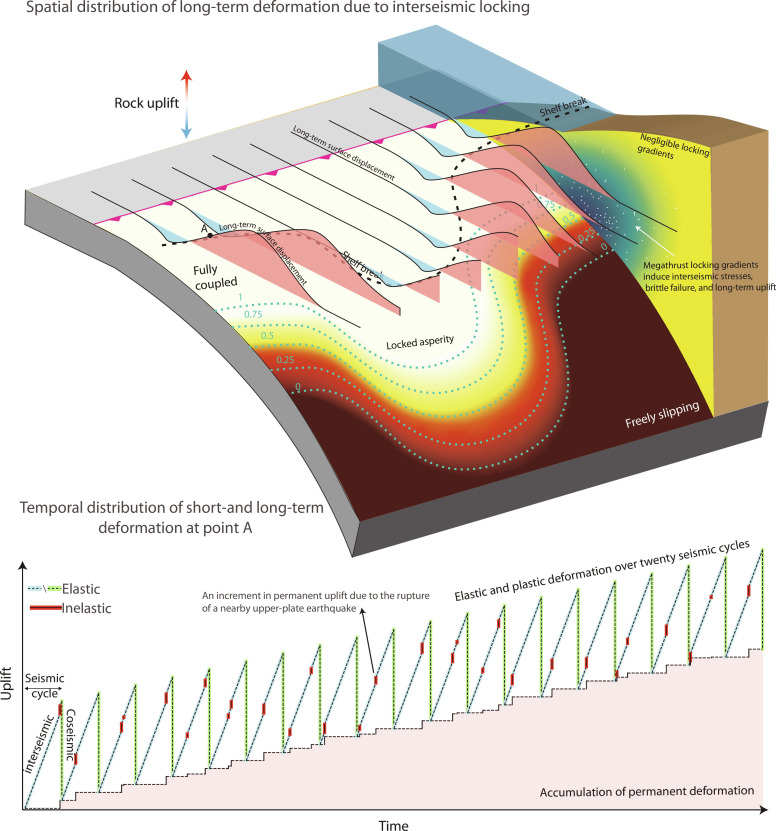
Illustration of megathrust locking imprinting subduction zone landscapes over many earthquake cycles. (**Top**) Spatial pattern of nonrecoverable deformation due to a coupling distribution along a subduction interface. White circles mark upper-plate interseismic seismicity activated by locking gradients. The total rock uplift from upper-plate earthquakes over many seismic cycles is depicted by 2D plots above the surface. (**Bottom**) Elastic and nonrecoverable deformation at point A during 20 seismic cycles.

Our model effectively explains the correlation between short-term and long-term deformation in subduction zones and indicates that incremental inelastic interseismic deformation accumulates over multiple seismic cycles, resulting in a long-term strain imbalance, and a coherent landscape signature (Fig. 5). This implies that to first order, the downdip pattern of megathrust locking tends to remain steady over landscape-shaping timescales (hundreds of kyr). If locking were to change frequently, subduction landscapes would integrate a fluctuating field of rock uplift, and the correlation between landscape and geodetically measured rock uplift would be lost. For example, the lumpy bathymetry and absence of striking slope break across the shelf edge at the Japan subduction stands in contrast to the regularity of the continental slope at the Cascadia and Central American subductions. This may illustrate the landscape signature of a shifting uplift pattern derived from frequent changes in megathrust coupling (*13*). Sedimentary series and marine terraces along the coastline of northeast Honshu show persistent subsidence at 10^3^- to 10^4^-year timescales but rock uplift at >10^5^ years, while the instrumented late interseismic phase records subsidence at the coastline (*26*, *63*–*65*). The varying deformation patterns and irregular topography likely reflect previous coupling configurations rather than the current interseismic deformation. The patterns of crustal deformation encoded in subduction landscapes over timescales from seconds to hundreds of kyr would, therefore, be an indirect but exploitable proxy for the evolution, stability, or transience of megathrust coupling over geological time and could be used to evaluate seismic hazard in regions with poor geodetic coverage.

## MATERIALS AND METHODS

### Interseismic seismicity in the forearc wedge

To model the distribution of seismicity throughout the forearc wedge we adopt a framework developed by Dieterich (*34*, *35*). This approach combines rate-and-state friction, fault mechanics, and statistical seismology to establish a constitutive relationship between stressing history and seismicity rate. It treats seismicity as a sequence of earthquake nucleation events adhering to time and stress-dependent, rate-and-state equations that characterize unstable slip. This framework has been widely used to describe the spatial and temporal distribution of seismicity that arises from changes in stress and stress rate and can thus be used to model aftershocks (*35*, *66*–*69*), tidal earthquake triggering (*70*, *71*), earthquake probabilities (*72*, *73*), and induced seismicity (*74*–*78*).

Under the above assumptions, *R*, the rate of seismicity, writes (*34*, *35*)

$$R=\frac{r}{{\dot{S}}_{b}\;\gamma };\dot{\gamma }=\frac{1-\gamma \dot{S}}{a\sigma }$$ (1)

where *r* is the background rate of seismicity, ${\dot{S}}_{b}$ is the background stress rate, γ is a state variable linking seismicity rate with time and stressing history (*34*), and *S* is the modified Coulomb stress

S=τ+(μ−α)σ (2)

In Eqs. 1 and 2, α and *a* are rate-and-state parameters relating changes in normal stress to friction and instantaneous slip rate to friction, respectively. σ and τ are the normal and shear stress acting on a population of earthquake sources, and μ is the static friction coefficient.

At steady state, γ evolves to $\gamma_{\text{ss}}=\frac{1}{\dot{S}}$ . It follows that the seismicity rate is proportional to

$$R_{\text{ss}}=\frac{r}{\dot{S_{b}}}\dot{S}$$ (3)

Assuming a constant background stressing rate across the forearc wedge, ${\dot{r}}_{b}$ , which leads to a steady seismicity rate, *r*, and considering that the Coulomb stress change rate remains constant during the interseismic period—therefore proportional to the Coulomb stress change—we can estimate the perturbed seismicity rate during the interseismic period at every point within the wedge by assessing the modified Coulomb stress change there

$$R_{\text{ss}}\left(x,z\right)\propto S\left(x,z\right)$$ (4)

#### Coulomb stress change across the forearc wedge

We consider a forearc wedge underlain by a megathrust in a homogeneous elastic half-space (fig. S1). We use the backslip framework (*5*) with Okada solutions (*37*, *38*) and calculate the interseismic strain in the forearc using planar dislocations that slip according to published slip deficit distributions along the megathrust interface (*8*, *22*, *28*). We use these geodetically derived slip deficit maps to determine where along the interface the coupling transitions from (i) coupled to partially slipping and further downdip to (ii) freely slipping.

We model the transition in slip rate between these two points by paving the interface with 100 rectangular dislocations whose slip varies linearly with downdip distance. We neglect 3D variations in coupling and extend these dislocations to a thousand kilometers in the along-strike direction. We also assume that the megathrust interface up-dip of point (i) is fully coupled because of the stress shadowing effect (*8*, *79*). We link strain and stress using Hooke’s law, assuming a shear modulus and Poisson’s ratio of 30 GPa and 0.25, respectively. Relying on this, we compute the interseismic Cauchy stress tensor along a triangular mesh that is offset by a kilometer from the megathrust interface, representing the upper plate. Last, assuming an Andersonian background stress state (*80*), we assume the forearc is populated with optimally oriented faults dipping at 30°, which we use for calculating the Coulomb stress change.

#### Permanent surface displacement from interseismic seismicity

We estimate the surface displacement from upper-plate interseismic seismicity by generating a synthetic earthquake catalog that represents multiple earthquake cycles and compute the associated surface displacements. We neglect lower plate seismicity because of its minor contribution to surface displacement (text S8 and fig. S11).

We calculate the Gutenberg-Richter distribution (*81*) for Cascadia, Chile, and the Himalayas by fitting the moment magnitude distribution of local seismic catalogs (*33*, *44*) and previous estimates of the local seismicity (*82*) and generate a random sequence of synthetic earthquakes whose magnitudes comply with the estimated *b* value (see texts S3 to S5 and figs. S3 to S7). We position the hypocenters of these synthetic events within a 3D domain so their location corresponds to the spatial distribution of seismicity according to Eq. 4. We do so using a sampling rejection algorithm, retaining earthquakes that occur at a random depth, *z*, and distance from the trench, *x* only if

$$\frac{S_{N}\left(x,z\right)}{\;\int_{c}^{1}S_{N}\left(x,z\right)}>u_{o}$$ (5)

where *u_o_* is a random number uniformly distributed between 0 and 1.

In Eq. 5, *S_N_*(*x*, *z*) is the modified Coulomb stress (Eq. 2) normalized with respect to the maximum modified Coulomb stress in the domain. We reduce computation time by limiting our randomly seeded hypocenters (*x*,*z*) to a possible rupture region where *S_N_* exceeds a small threshold *c* of 5% (see text S7 and figs. S9 and S10 for verification of model parameters). The along-strike position of these events is uniformly distributed within the domain.

We compute the surface displacement imparted by the rupture of all the synthetic events by assuming they occur on rectangular faults. This is achieved using the Okada dislocation model (*37*, *38*), and empirically derived relations between moment magnitude (*M*_w_), along-strike rupture length (*L*_r_), and downdip extent *D*_r_ to determine the rupture area A (=*L*_r_ · *D*_r_) for each event (*36*)

$$L_{r}=10^{\frac{M_{w}-4.38}{1.49}};D_{r}=10^{\frac{M_{w}-4.06}{2.25}}$$ (6)

The events’ slip (*s*) is then obtained from its seismic moment as

$$s=\frac{10^{1.5M_{w}+9.05}}{A\cdot G}$$ (7)

where *G* is the shear modulus. We consider that events nucleate on 30°-dipping thrust faults (fig. S1), which are equally likely to dip toward the trench (seaward) or away from it (landward). We assume earthquakes rupture updip from our guessed hypocenter and reject earthquakes whose updip rupture length extends below the megathrust or above the surface. We also impose that earthquakes are 95% less likely to rupture within the shallowest 2 km of the forearc, in accordance with the lack of shallow seismicity observed globally in this depth range (*9*).

We continue to generate synthetic events and sum their imparted surface displacements in an iterative fashion until

$$\frac{\sigma_{v}^{\text{max}}\left(x\right)}{v_{\text{mean}}\left(x\right)}<c_{0}$$ (8)

where *v*_mean_(*x*) is the cumulative vertical displacement averaged along strike measured at distance *x* from the trench (fig. S1), $\sigma_{v}^{\max}$ (*x*) is the maximum along-strike SD of the cumulative vertical displacement, and *c*_0_ is a threshold set to 0.2. We convert rock uplift to uplift rate by dividing the cumulative vertical displacement by the recurrence time of the randomly seeded earthquakes, which we infer from the *a* value of the Gutenberg-Richter distribution. For more details regarding the quantification of our estimated uplift rates and on how we incorporate the dimensions of our 3D domain in this process, please refer to text S9.

It is important to note that we limit the maximum magnitude according to the largest *D_l_* capable of fitting in the rupture zone (fig. S1) and set the minimum magnitude to 4 as smaller earthquakes produce negligible surface displacement (text S2 and fig. S2) for the *b* values typically measured in convergent contexts (*83*). Last, we determine the along-strike extent of the domain according to the maximum earthquake length (fig. S1). For cases where we vary the locking distance from the fault systematically (e.g., Fig. 3), we set the *b* value to 0.9 according to a global complication of thrust events (*83*). As we are only interested in the spatial pattern of the uplift profile in these cases, we normalize the surface uplift with respect to the maximum value when averaged along strike. For the case shown in Fig. 3C, we compute the location of synthetic earthquakes along 800 5-km-long domains with varying coupling and then compute the surface displacement imparted by earthquakes registered in all domains along a 4000-km-long region.

#### Northern Chile long-term uplift shape derived from topography and river incision

The coastal region of northern Chile (~18°S to 25°S) is an extremely arid region with precipitation rates well below 100 mm/year (*84*). The main rivers flow from the Andes and dissect the landscape of the coastal range during rare extreme flooding events (*85*). The coastal range catchments are often perched above the traversing channels and have very low basin-averaged denudation rates (<0.05 mm/year) suggesting that the equilibration time of these tributary river networks is well over millions of years (*84*, *86*, *87*). This very slow response time, combined with an extreme-event-dominated incision limits the use of the fluvial landscape to estimate long-term uplift signature with traditional tools such as channel steepness and the stream power incision (*15*, *88*). Fortunately, the presence of the larger Andean rivers crossing the arid coastal range allows us to use the difference between these river profiles and the uplifted and warped topography as a proxy for the regional uplift pattern.

To do so, we focus our analysis south of the outlet of Rio Loa (21°25′S), one of the few mainland rivers connected to the ocean in northern Chile. This region is characterized by fairly uniform lithology (*89*) so changes in river incision and surface elevation cannot be attributed to variations in rock erodibility. We use lsdtopytools flow routines (*90*) to extract the main river channel from ALOS World 30-m digital elevation model (*91*) and analyze its profile. We constrain incision along the river by measuring the relief between the river bed and the incised surface flanking the canyon in a 1.5-km window across the flow direction (Fig. 2C1). Local relief increases sharply in the immediate proximity of the river and barely changes beyond the canyon walls. This supports the hypothesis that (i) recent river incision does not shape the landscape beyond the river valley, (ii) the river transports the sediment flux to the ocean without intermediate deposition over large areas, and (iii) the uplifted surface can be used as a passive strain marker. Furthermore, we extract a 120-km-wide W-E topographic swath profile, south of the Rio Loa where its influence is negligible. We use the variation in elevation along the swath profile, measured from a base level situated at a plateau between the coastal range and the cordillera, as a second indicator for uplift. The resemblance between the two independent measurements supports the use of the regional topography as a proxy for long-term uplift.

## Supplementary Materials

### This PDF file includes:

Supplementary Text S1 to S11 Figs. S1 to S13 Table S1 References
