# Supplementary material for: Megathrust locking encoded in subduction landscapes
Source: Sci Adv. 2024 Apr 26;10(17):eadl4286. doi: 10.1126/sciadv.adl4286 (PMC11051668; doi:10.1126/sciadv.adl4286)
Supplement: Supplementary file 1 — Supplementary Text S1 to S11 Figs. S1 to S13 Table S1 References [file sciadv.adl4286_sm.pdf]

Science Advances
AAAS

Supplementary Materials for

Megathrust locking encoded in subduction landscapes

Bar Oryan *et al.*

Corresponding author: Bar Oryan, bar.oryan@columbia.edu

Sci. Adv. **10**, eadl4286 (2024)
DOI: 10.1126/sciadv.adl4286

This PDF file includes:

Supplementary Text S1 to S11
Figs. S1 to S13
Table S1
References

Text S1 - Domain setup:

Please see the figure below for an illustration of our model setup. Please note that the domain surface uplift and mean along strike uplift field are examples of what an uplift field might look like.

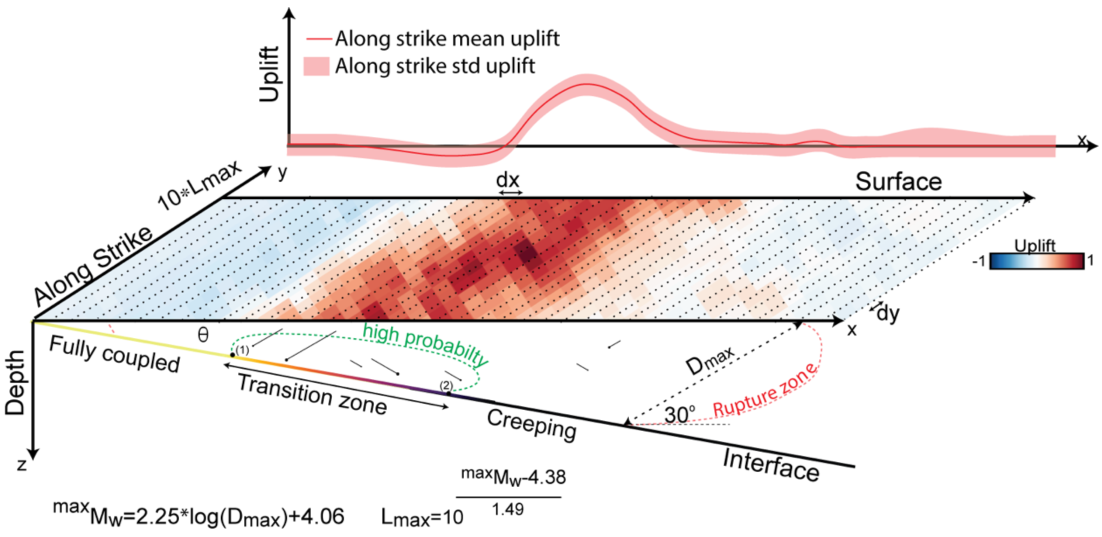

Figure S1 - Illustration of domain dimensions, synthetic earthquake maximum rupture properties, and model setup and results. Rupture region denotes the area where synthetic earthquakes are first guessed and correspond to where S_N (See equation 5) exceeds a small threshold c. High probability zone marks a region where synthetic earthquake density is high, and most surface displacement is concentrated. Dots and adjacent lines mark nucleation points and faults on which earthquakes rupture. Dots on the surface show positions where we compute surface displacement and are color-coded by an example uplift field. D_{max}, ${}^{max}M_w$ and L_{max} are the synthetic earthquake maximum values for the following (1) along dip rupture length (2) moment magnitude and, (3) along strike rupture length, respectively. The interface is color-coded by its coupling.

Text S2 - Sampling the Gutenberg Richter distribution

The moment magnitude of synthetic events is drawn from the truncated Gutenberg Richter distribution (*93*) given a certain b value and maximum and minimum event sizes. The upper limit for event size is set according to the dimension of the forearc (Fig. S1), and the minimum size is set due to computational constraints. For typical b values (~0.9-1) small events do not generate substantial surface displacement but pose a considerable computational task. This point is illustrated below, where uplift drawn from the Gutenberg Richter distribution using b value of 0.9 generate self-similar uplift pattern (Fig. S2B) and the total uplift from Mw>3 is essentially identical to events Mw>4 (Fig. S3A). Therefore, we use a minimum event size of 4 in the manuscript.

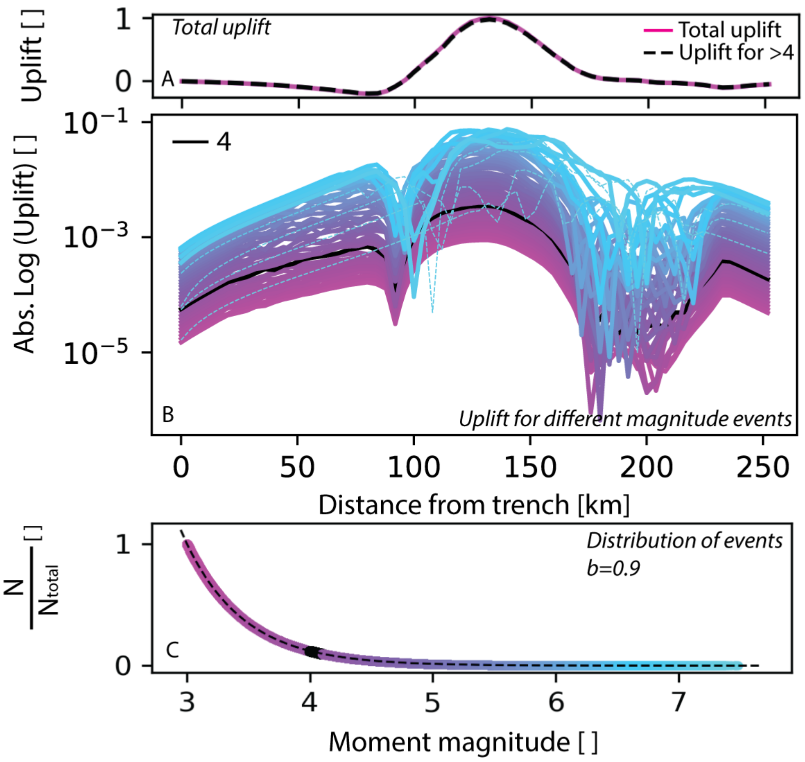

Figure S2 - Inelastic surface uplift for various earthquake magnitudes. A - Magenta and dashed black lines show the along strike normalized mean uplift for all and larger than 4 Mw earthquakes, respectively. B - Uplift for events grouped by their magnitude illustrated by the colors shown in panel C. Uplift for magnitude >7 and magnitude 4 events are marked with a thin dash and a black line, respectively. C - Earthquake magnitudes populated in the domain for a random sample of the truncated Gutenberg Richter distribution where b=0.9 and the minimum and maximum magnitude are 3 and 7.5, respectively (see Figure S1). For a full description of model parameters, see Table S1.

Text S3 - Cascadia

Our study specifically targeted the southern and central sections of the Cascadia subduction zone, where there is extensive coverage of local seismicity (*33*) and available datasets on interseismic uplift (Fig. S3). To exclude the effects of along-strike 3D changes in the subduction zone geometry and slow slip events, we chose not to extend our transect into the northern section of the Cascadia subduction zone. We used the reported Richter's local magnitude in the catalog we employed (*33*) and estimated the moment magnitude (*94*).We then fitted a and b values assuming a Gutenberg Richter distribution using the least square method (Fig S4). We only consider seismicity recorded in the upper plate and note that the less-than-optimal fit is due to the rolling nature of the deployment of the OBS array (*33*). We would like to highlight that we only use a local catalog recorded by an array of ocean-bottom seismometers (OBS) as we are interested in the precise position of microseismicity offshore. Finally, the long-term uplift uncertainty (Fig. 2A1) stems from both the standard deviation produced by our model (Eq. 8) and the uncertainty produced by the fit (Fig. S4).

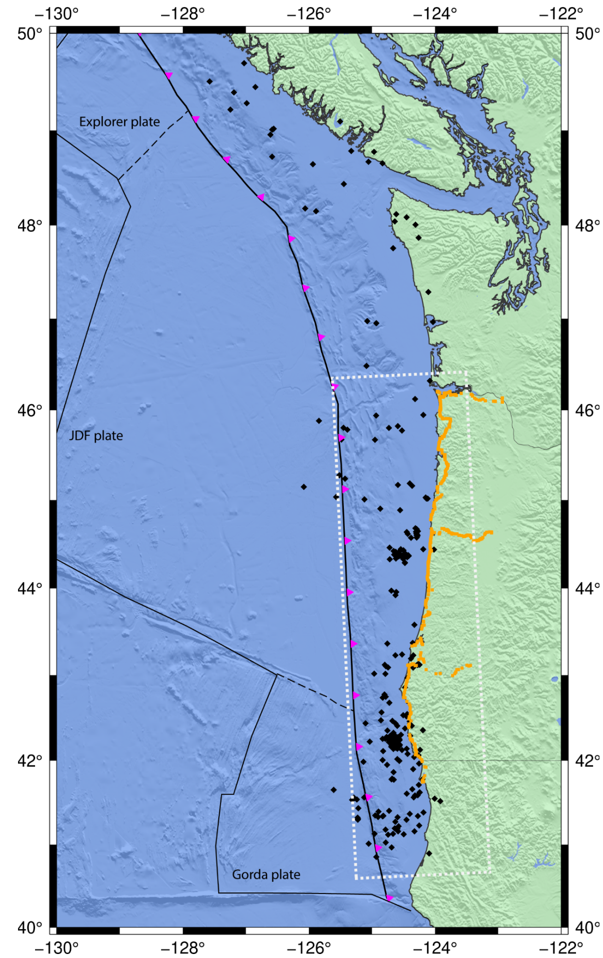

Figure S3 - Map of data used for the Cascadia case. Black diamonds mark recorded seismicity (*33*). Orange dots represent the position of leveling data (*92*). Black line with magenta triangles shows the location of the trench. Dash white rectangle denotes the cross-section shown in Fig 2.

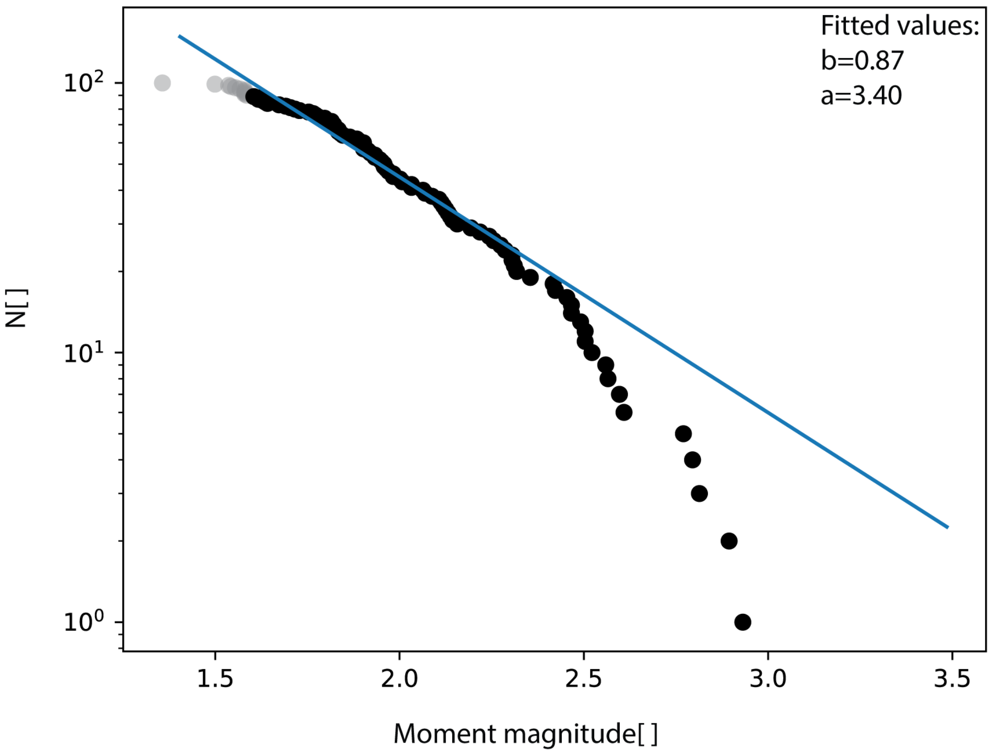

Figure S4 -Upper plate seismicity (*33*) **used to constrain a and b values.** Grey and black circles represent events below and above the estimated completeness magnitude, respectively. Only the latter events were used in fitting the data. The blue curve marks the Gutenberg Richter distribution corresponding to the fitted a and b parameters.

Text S4 - Himalayas

We focused on the central section of the Himalayas, where ample evidence for interseismic deformation and long-term uplift exists (Fig S5). Our region of interest is characterized by a and b values of -5.3 (km^2yr^{-1}) and 1.06, respectively (*82*). As we are only interested in upper plate seismicity, we account for the ratio of upper plate events (*42*, *43*) to the total seismicity observed in our transect (Fig. 2B4). This ratio is similar to earlier estimations observed between the productivity along the Main Himalayan Thrust and off-interface seismicity (*95*, *96*). Finally, our long-term uplift uncertainty (Fig. 2B1) is the sum of the standard deviation produced by our model (Eq. 8) and the uncertainty in a-value (*95*).

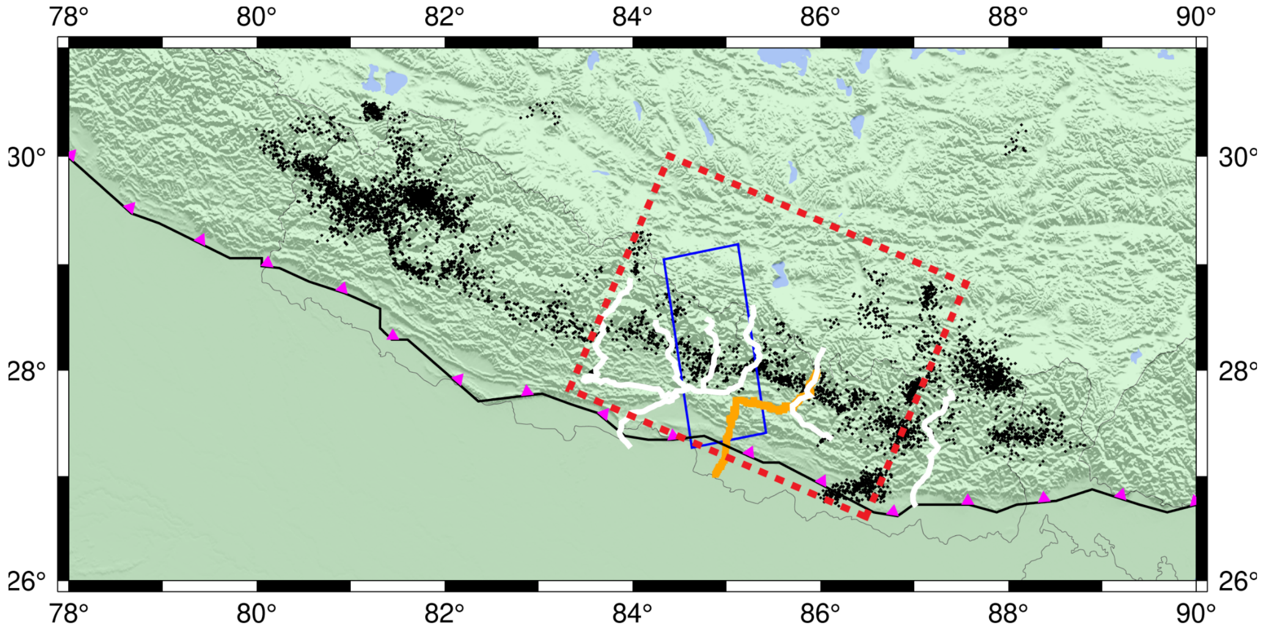

Figure S5 - Map of data used for the Himalayas case. Black points mark recorded (*42*, *43*). Orange line and blue rectangle show leveling data transect (*17*) and ALOS track (*18*), respectively. White curves denote rivers used to constrain fluvial incision rates(*14*). Red dash rectangle denotes the cross-section area (Fig. 2B4). Black line with magenta triangles shows the location of the Main Himalayan Thrust.

Text S5 - Chile

We targeted a region in northern Chile (Fig. S6) where seismicity is not predominantly linked to specific faults as further north shallow seismic activity is associated with the Adamito Fault (*44*). We used the reported local magnitude in the catalog (*44*) and converted it to moment magnitude (*94*). We then fitted a and b values assuming a Gutenberg Richter distribution using the least square method (Fig S7).

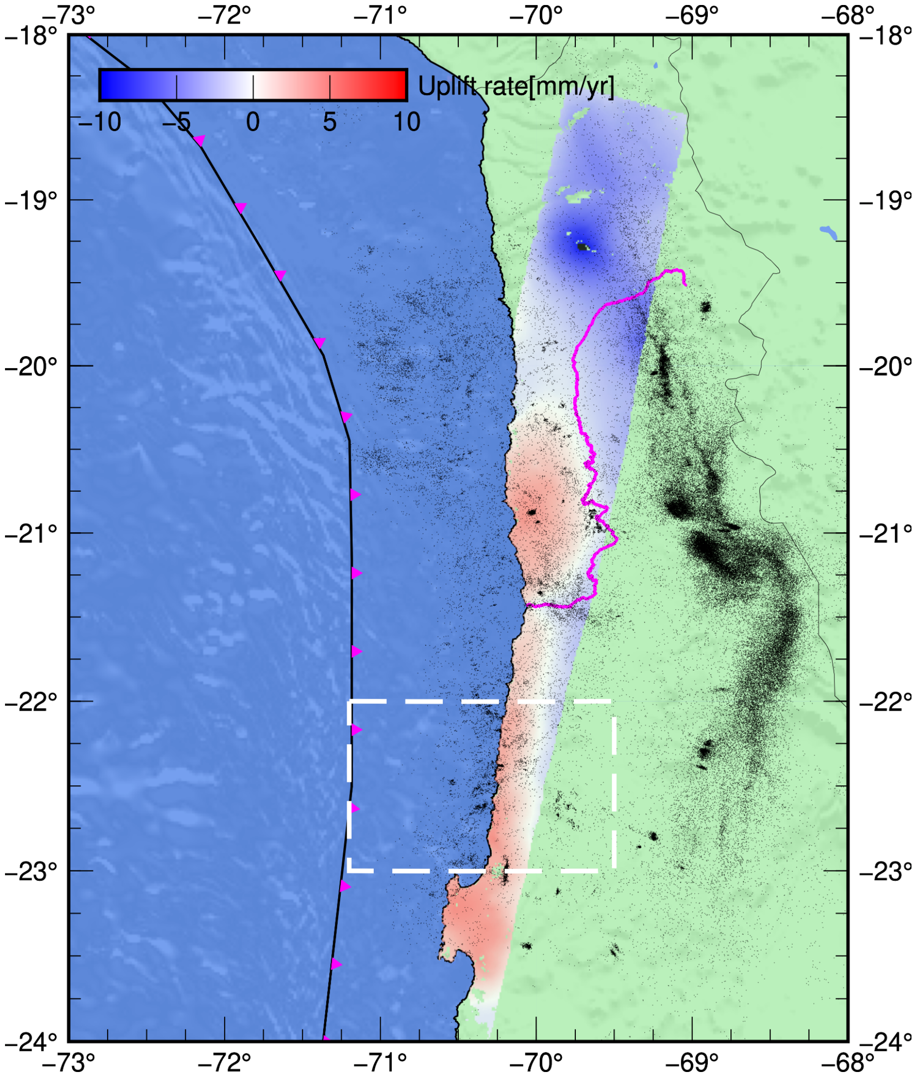

Figure S6 - Map of data used for the northern Chile case. Black points mark recorded seismicity (*44*). Intersesismic uplift rates recorded by Envisat are shown by blue and red colors (*22*). Magenta curve denotes the river used to constrain the uplift shape(See method section). White dash rectangle denotes the area of the cross-section (Fig. 2C4). Black line with magenta triangles shows the location of the trench.

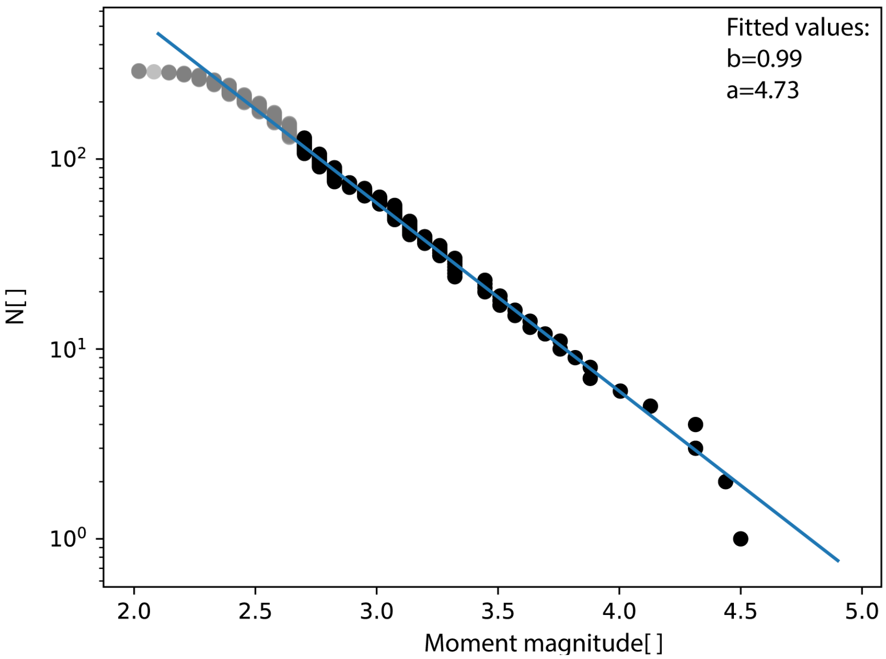

Figure S7 - Upper plate seismicity (*44*) **used to constrain a and b values.** Grey and black circles represent events below and above the estimated completeness magnitude, respectively. Only the latter events were used in fitting the data. The blue curve marks the Gutenberg Richter distribution corresponding to the fitted a and b parameters.

Text S6 - Agreement between our model and recorded seismicity

We compare the spatial distribution of recorded seismicity in Cascadia, Himalayas and northern Chile with generated synthetic events derived by our method (eq. 5). For reference, we also include synthetic events that are uniformly distributed within the forearc. For simplicity, we assume these events all have a moment magnitude of 4.

To capture the variability in the recorded seismicity, we assume a 5km uncertainty in the position of documented events and sample 1000 occurrences for each earthquake, considering a normal distribution. We emphasize that seismic activity in northern Chile and Cascadia is limited due to the arrangement of seismometers used to capture these events. In Cascadia, on-shore seismicity is missing due to the OBS array located off-shore, while in Chile, events close to the trench are absent due to the position of the land-based array. Nonetheless, given these limitations and the fact that recorded seismicity is documented in a time span of a few years, merely a fraction of one interseismic period suggests a good correlation with our synthetic events (Fig S8), particularly along the x-axis where peak seismicity coincides nicely.

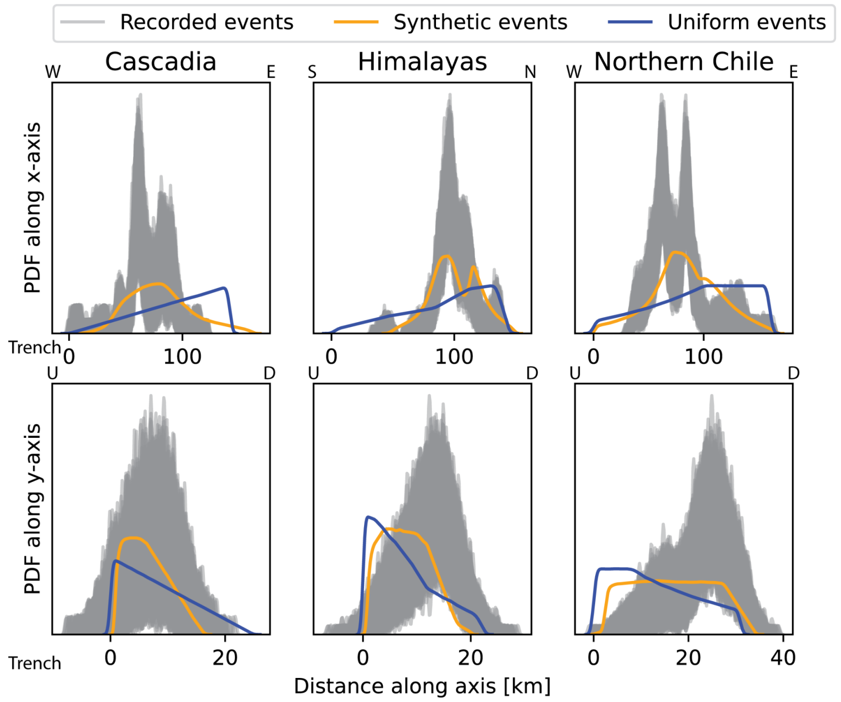

Figure S8 - Comparison between synthetic events (Eq. 5; orange), uniform distribution events (blue), and upper plate seismicity (*33*, *42–44*)**.** Each gray line corresponds to one sample generated from the normal distribution. The orange and blue curves correspond to the PDF derived from 1 million events.

Text S7 - Verification of model parameters

We conducted an extensive exploration of model parameters to assess the robustness of our key findings. Firstly, we investigated the impact of varying the width of the transition zone on the pattern of inelastic uplift. As anticipated, the location of the hinge line remained relatively constant, while the width of the inelastic uplift zone exhibited a correlation with the width of the elastic transition zone (Fig. S9A). When testing the effect of the dip angle, we observed that larger dip angle values resulted in the hinge line moving closer to the trench and the inelastic uplift zone widens (Fig. S7C). This is likely the result of the interaction between the dip angle, free surface, and locking gradients stresses. Next, we systematically adjusted the cutoff parameter (c in eq. 5) and observed that while the hinge line remained stationary, the width of the uplift zone decreased with larger cutoff values (Figs. S9B & S10). This is attributed to synthetic earthquakes and resulting deformation concentrated in the region of the highest stresses near the downdip end of the coupled zone.

It is interesting to note that increasing the cutoff parameter is similar to assuming that large regions of the upper plate are far from compressional yield. As can be seen in Figs. S9B and S10, this does not fundamentally alter our results, as we still maintain a strong hinge line with a pronounced permanent uplift region. This suggests that as long as a small part of the upper plate is in compression, a characteristic landscape (as described in the main text) will be produced.

Lastly, varying the alpha parameter, which is the least constrained in our model, showed minimal impact on the width of the uplift zone, and the position of the hinge line remained largely constant (Fig. S9D).

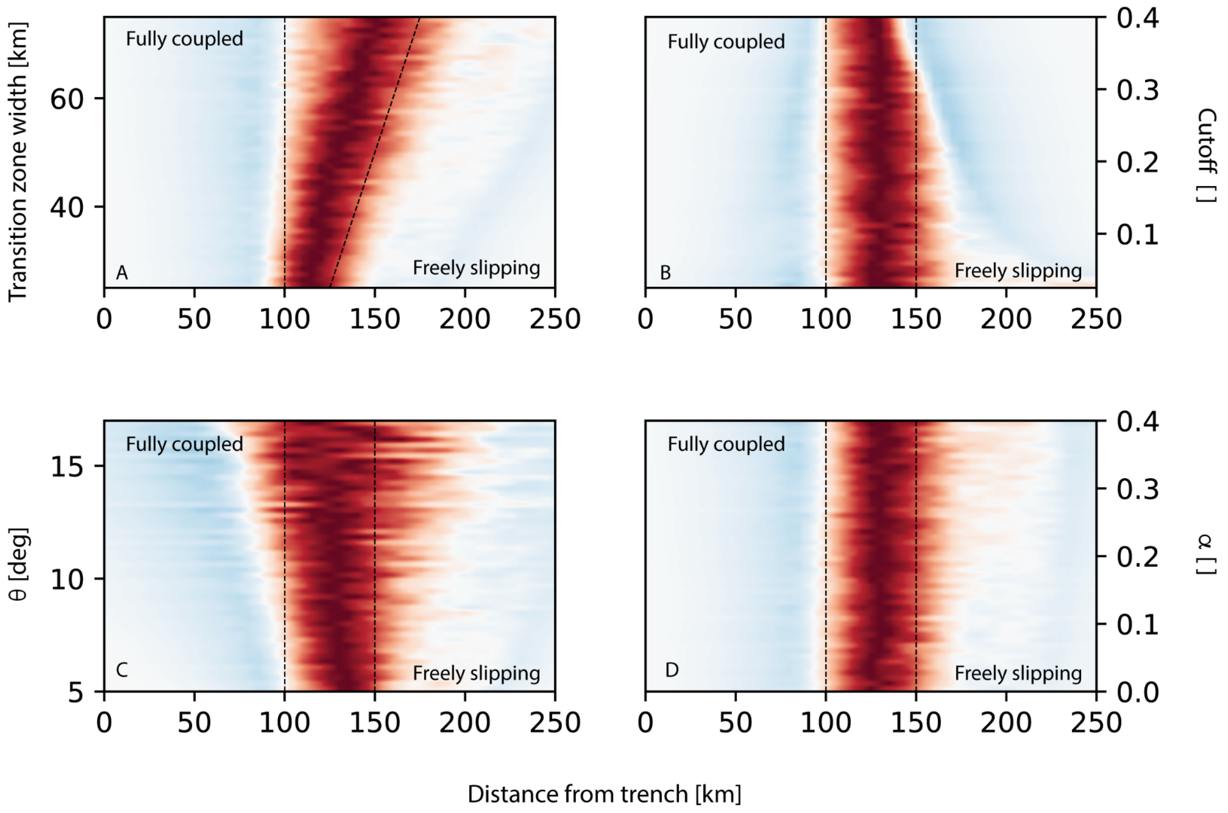

Figure S9 – Long-term uplift for different parameters. Dash thin lines mark the transition in coupling. For a full description of model parameters, see Table S1.

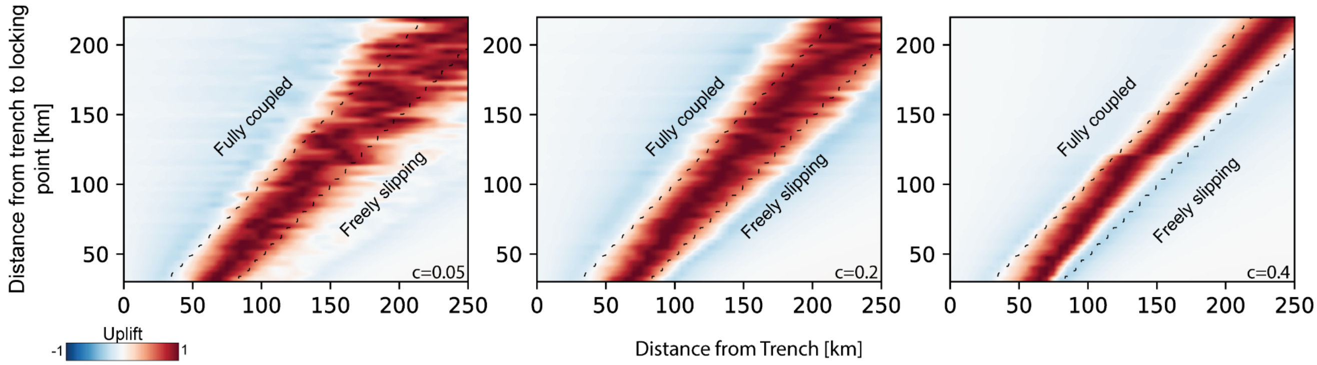

Figure S10 - Mean surface inelastic uplift for varying cut-off parameters. Dash thin lines mark the transition in coupling. For a full description of model parameters, see Table S1.

Text S8 -The contribution of an inelastic lower plate to long-term surface uplift

To reduce computation time, we ignored the contribution of inelasticity in the lower plate (LP) even though LP earthquakes are documented in close proximity to the down dip end of the fully coupled zone (Figs. 2A4,2B4 & 2C4). Here we demonstrate that the effect of LP seismicity resulting from locking gradients on surface deformation is negligible. We consider a forearc fully locked for a distance of 100km and assume that the background stress state of the LP and upper plate (UP) is extensional (*10*) and compressional, respectively. We compute the extensional coulomb stress change in the LP and the compressional coulomb stress change in the UP (Fig S11B), and populate the plates with synthetic earthquakes according to equation 5. We consider LP and UP earthquakes to nucleate on 60º-dipping normal faults and 30º-dipping thrust faults relative to the horizon, respectively. These events are equally likely to dip toward the trench (seaward) or away from it (landward).

We sum the surface displacement of these synthetic events and observe that the depth at which LP earthquakes occur limits their influence on the surface. This leads to a relatively modest LP long-term displacement, which is minor in comparison to the contribution of shallower UP thrust events (Fig. S11A). We highlight that while this analysis examines a specific coupling configuration, it points to a broader phenomenon demonstrating that down-going inelastic seismicity resulting from locking gradients has a small effect on the surface.

Finally, for completeness, we also show that assuming a compressional LP (Fig. S11C) results in an even smaller long-term uplift concentrated in the vicinity of the trench, having no real impact on the principal uplift field situated above the transition zone (Fig. S11A).

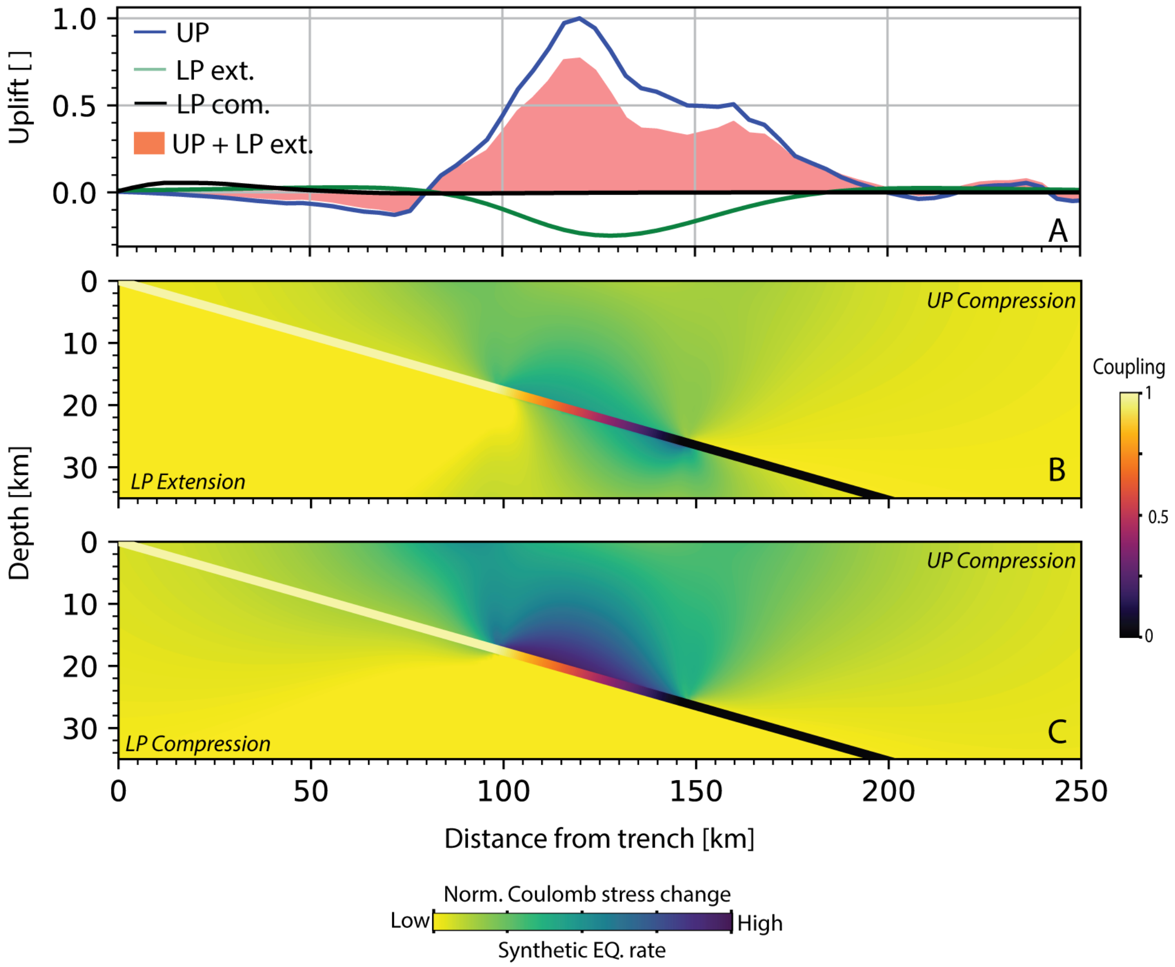

Figure S11 - Contribution of lower plate yielding to permanent surface displacement. A - Uplift from compressional and extensional UP and LP. B - Normalized UP Compressional and LP extensional coulomb stress change. C - Normalized UP and LP Compressional and coulomb stress change. Coulomb stress change values are normalized with respect to the largest absolute value in the domain. The interface is color-coded by the coupling model used. For a full description of model parameters, see Table S1.

Text S9 - Converting long-term surface uplift to uplift rate

Seismicity is recorded in a given forearc within a surface area A_f during time T, yielding Gutenberg Richter distribution with a and b values. The number of earthquakes larger than M_w 4 during a certain unit of time and area is N_f:

$$N_f = \frac{10^{a-4b}}{T \cdot A_f}$$

Using our model, we compute the long-term surface displacement ,$U(x, y)$, within a domain with a surface area A_d resulting from N_d synthetic events randomly sampled from a Gutenberg Richter distribution with the same measured forearc b-value. The uplift rate, $V_u(x, y)$, is then:

$$V_u(x,y) = U(x,y) \cdot \; N_f / \frac{N_d}{A_d} = U(x,y) \cdot \frac{10^{a-4b} \cdot A_d}{A_f \cdot T \cdot N_d}$$

Where x and y is distance from the trench and along strike, respectively.

Text S10 – Sensitivity of long-term uplift to synthetic fault geometries

We test the sensitivity of long-term uplift pattern on the dip angle of synthetic events. We test four scenarios where dip angles are (1) randomly sampled from a normal distribution of mean of 30° and standard deviation of 10°, (2) 20°, (3) 30° (used in the main text) and (4) 40°. As can be seen (Fig. S12) the dip angle does not have a significant effect on the shape of uplift. This is because faults are equally likely to extend in both the seaward and landward directions and so the overall pattern remains largely consistent.

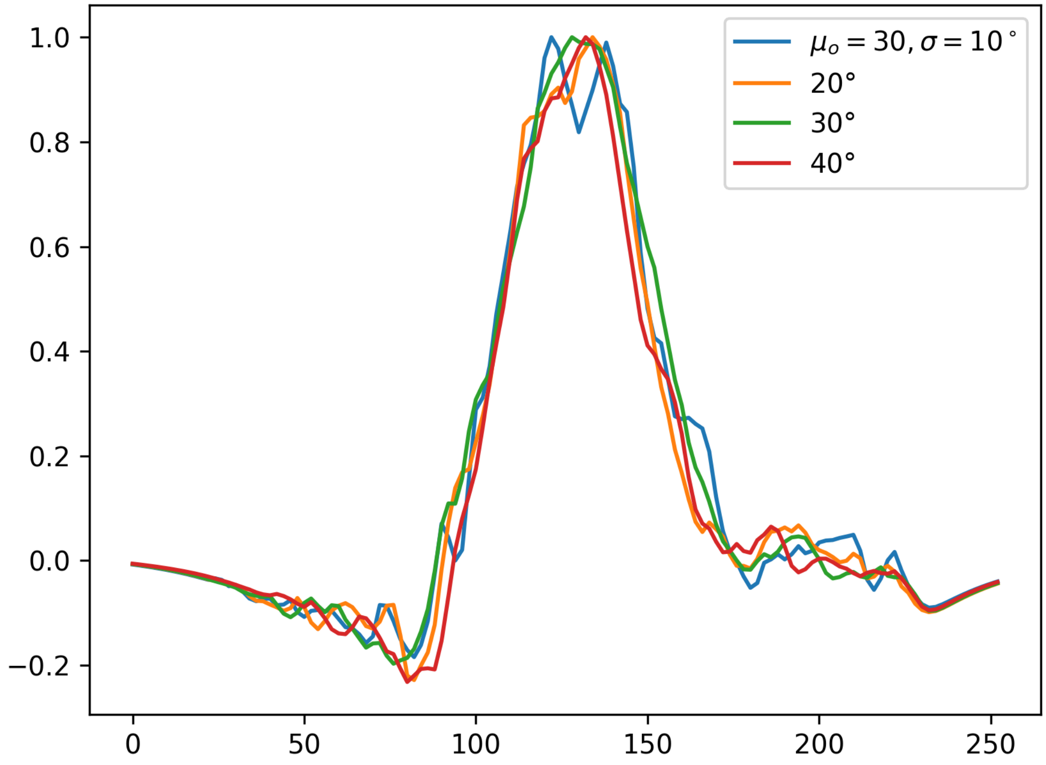

Figure S12 - Sensitivity of long-term uplift pattern to synthetic dip angle. Normalized uplift pattern for four different assumed dip angles for 100K synthetic receiver faults. For a full description of model parameters, see Table S1.

Text S11 – Focal mechanisms of recorded earthquakes in Cascadia, the Himalayas, and Chile

To examine our assumption regarding the stress orientation of the upper plate, we inspected the focal mechanisms of earthquakes in Cascadia, the Himalayas, and Chile as recorded by the Global Centroid Moment Tensor (GCMT) catalog (*97*, *98*). We focus on recorded CMT dip angles, reporting an average of 35.3 degrees and a magnitude of 5.3 for 162 earthquakes across these three active margins (Fig. S3). This aligns with our hypothesized Andersonian stress state in the upper plate, where dip angles are presumed to be rotated to an angle of 30 degrees relative to the horizontal. A particularly good correlation is found in Chile (Fig. S13). Dip angles in the Himalayas show a decent correlation, especially in the upper plate, which is our main area of interest. Cascadia, however, exhibits a weaker correlation, but the sample set there is limited to only three events and likely does not offer a comprehensive representation of stress orientation in the Cascadia upper plate. Lastly, we note that the GCMT catalog presents two possible solutions for the dip angle, but we only examine the first because our long-term uplift solution already accounts for both possible orientations.

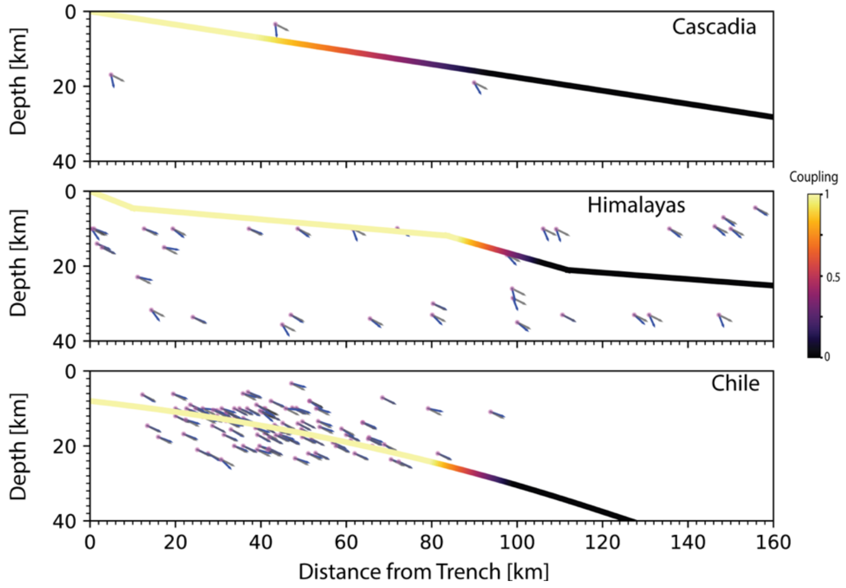

Figure S13 - Dip angle of earthquakes in Cascadia, the Himalayas, and Chile. Magenta points denote the earthquakes' hypocenter. Blue line marks the dip angle documented by the GCMT catalog (*97*, *98*). Gray line show a dip angle of 30°. Subduction zone interfaces are color-coded by the coupling model used (e.g., Fig. 2).

Figure	θ [deg]	α [ ]	C [ ]	Horizontal Distance between the trench and downdip of locked zone [km]	Transition zone width [km]
2A Cascadia	10 (*99*)	0.2*	0.05	39.6	59.7
2B Himalayas	Varying (*28*)	0.2	0.05	86.5	19.9
2C Chile	Varying (*99*)	0.2	0.05	80.3	20.0
3	7**	0.2	0.05	30-205	50
4	7	0.2	0.05	65-90	50
S2	10	0.2	0.05	100	50
S9A	7	0.2	0.05	100	25-75
S9B	7	0.2	0.025-0.4	100	50
S9C	5-17	0.2	0.05	100	50
S9D	7	0-0.4	0.05	100	50
S10	7	0.2	0.05-0.4	30-205	50
S11	7	0.2	0.05	100	50
S12	7	0.2	0.05	100	50

Table S1 - Model parameters presented in the main text and Supplementary information.
* Based on the suggested value in the literature (*34*).
**Corresponds to the average dip angle of forearcs examined by Malatesta et al.(*13*).

REFERENCES AND NOTES

1. H . Lay, C. J. Kanamori, K. D. Ammon, A. R. Koper, L. Hutko, H. Ye, T. M. Yue, Depth-varying rupture properties of subduction zone megathrust faults. *J. Geophys. Res. Solid Earth* **117**, 1–21 (2012).
2. E . A. Wirth, V. J. Sahakian, L. M. Wallace, D. Melnick, The occurrence and hazards of great subduction zone earthquakes. *Nat. Rev. Earth Environ.* **3**, 125–140 (2022).
3. K. Wang, Y. Hu, J. He, Deformation cycles of subduction earthquakes in a viscoelastic Earth. *Nature* **484**, 327–332 (2012).
4. R. Burgmann, M. G. Kogan, G. M. Steblov, G. Hilley, V. E. Levin, E. Apel, Interseismic coupling and asperity distribution along the Kamchatka subduction zone. *J. Geophys. Res. Solid Earth* **110**, (2005).
5. J. C. Savage, A dislocation model of strain accumulation and release at a subduction zone. *J. Geophys. Res.* **88**, 4984–4996 (1983).
6. B. W. Tichelaar, L. J. Ruff, Depth of seismic coupling along subduction zones. *J. Geophys. Res. Solid Earth* **98**, 2017–2037 (1993).
7. E . M. Hill, J. C. Borrero, Z. Huang, Q. Qiu, P. Banerjee, D. H. Natawidjaja, P. Elosegui, H. M. Fritz, B. W. Suwargadi, I. R. Pranantyo, L. Li, K. A. Macpherson, V. Skanavis, C. E. Synolakis, K. Sieh, The 2010 Mw7.8 Mentawai earthquake: Very shallow source of a rare tsunami earthquake determined from tsunami field survey and near-field GPS data.
Earth **117**, 159 (2012).
8. E . O. Lindsey, R. Mallick, J. A. Hubbard, K. E. Bradley, R. V. Almeida, J. D. P. Moore, R. Burgmann, E. M. Hill, Slip rate deficit and earthquake potential on shallow megathrusts. *Nat. Geosci.* **14**, 321–326 (2021).
9. C . H. Scholz, Earthquakes and friction laws. *Nature* **391**, 37–42 (1998).
10. B. Oryan, W. R. Buck, Larger tsunamis from megathrust earthquakes where slab dip is reduced. *Nat. Geosci.* **13**, 319–324 (2020).
11. R. E. Wells, R. J. Blakely, Y. Sugiyama, D. W. Scholl, P. A. Dinterman, Basin-centered asperities in great subduction zone earthquakes: A link between slip, subsidence, and subduction erosion? *J. Geophys. Res.* **108**, 2072 (2003).
12. T .-R. A. Song, M. Simons, Large Trench-Parallel Gravity Variations Predict Seismogenic Behavior in Subduction Zones. *Science* **301**, 630–633 (2003).
13. L . C. Malatesta, L. Bruhat, N. J. Finnegan, J.-A. L. Olive, Co-location of the Downdip End of Seismic Coupling and the Continental Shelf Break. *Earth* **126**, e2020JB019589 (2021).
14. J. Lave, J. P. Avouac, Fluvial incision and tectonic uplift across the Himalayas of central Nepal. *J. Geophys. Res. Solid Earth* **106**, 26561–26591 (2001).
15. B. J. Meade, The signature of an unbalanced earthquake cycle in Himalayan topography? *Geology* **38**, 987–990 (2010).
16. F. J. Clubb, S. M. Mudd, T. F. Schildgen, P. A. van der Beek, R. Devrani, H. D. Sinclair, Himalayan valley-floor widths controlled by tectonically driven exhumation. *Nat. Geosci.* **16**, 739–746 (2023).
17. M. Jackson, R. Bilham, Constraints on Himalayan deformation inferred from vertical velocity fields in Nepal and Tibet. *J. Geophys. Res. Solid Earth* **99**, 13897–13912 (1994).
18. K. M. Sreejith, P. S. Sunil, R. Agrawal, A. P. Saji, A. S. Rajawat, D. S. Ramesh, Audit of stored strain energy and extent of future earthquake rupture in central Himalaya. *Sci. Rep.* **8**, 16697 (2018).

19. B. Rousset, C. Lasserre, N. Cubas, S. Graham, M. Radiguet, C. DeMets, A. Socquet, M. Campillo, V. Kostoglodov, E. Cabral-Cano, N. Cotte, A. Walpersdorf, Lateral Variations of Interplate Coupling along the Mexican Subduction Interface: Relationships with Long-Term Morphology and Fault Zone Mechanical Properties. *Pure Appl. Geophys.* **173**, 3467–3486 (2016).
20. M. Saillard, L. Audin, B. Rousset, J.-P. Avouac, M. Chlieh, S. R. Hall, L. Husson, D. L. Farber, From the seismic cycle to long-term deformation: linking seismic coupling and Quaternary coastal geomorphology along the Andean megathrust. *Tectonics* **36**, 241–256 (2017).
21. D . Melnick, Rise of the central Andean coast by earthquakes straddling the Moho. *Nat. Geosci.* **9**, 401–407 (2016).
22. R. Jolivet, M. Simons, Z. Duputel, J. Olive, H. S. Bhat, Q. Bletery, Interseismic Loading of Subduction Megathrust Drives Long-Term Uplift in Northern Chile. *Res. Lett.* **47**, e2019GL085377 (2020).
23. A. Madella, T. A. Ehlers, Contribution of background seismicity to forearc uplift. *Nat. Geosci.* **14**, 620–625 (2021).
24. C . W. Baden, D. L. Shuster, F. Aron, J. C. Fosdick, R. Burgmann, G. E. Hilley, Bridging earthquakes and mountain building in the Santa Cruz Mountains, CA. *Sci. Adv.* **8**, eabi6031 (2022).
25. G. C. P. King, R. S. Stein, J. B. Rundle, The growth of geological structures by repeated earthquakes 1. conceptual framework. *Earth* **93**, 13307–13318 (1988).
26. T . Nishimura, Pre-, Co-, and Post-Seismic Deformation of the 2011 Tohoku-Oki earthquake and its implication to a Paradox in Short-Term and Long-Term Deformation. *J. Disaster Res.* **9**, 294–302 (2014).
27. V . Mouslopoulou, O. Oncken, S. Hainzl, A. Nicol, Uplift rate transients at subduction margins due to earthquake clustering. *Tectonics* **35**, 2370–2384 (2016).
28. J. R. Elliott, R. Jolivet, P. J. Gonzalez, J.-P. Avouac, J. Hollingsworth, M. P. Searle, V. L. Stevens, Himalayan megathrust geometry and relation to topography revealed by the Gorkha earthquake. *Nat. Geosci.* **9**, 174–180 (2016).
29. B. Oryan, P. M. Betka, M. S. Steckler, S. L. Nooner, E. O. Lindsey, D. Mondal, A. M. Mathews, S. H. Akhter, S. Singha, O. Than, New GNSS and Geological Data From the Indo-Burman Subduction Zone Indicate Active Convergence on Both a Locked Megathrust and the Kabaw Fault. *JGR Solid Earth* **128**, e2022JB025550 (2023).
30. M. S. Paterson, T. Wong, *Experimental Rock Deformation — The Brittle Field* (Springer-Verlag, 2005).
31. R. Cattin, J. P. Avouac, Modeling mountain building and the seismic cycle in the Himalaya of Nepal. *J. Geophys. Res. Solid Earth* **105**, 13389–13407 (2000).
32. D . Davis, J. Suppe, F. A. Dahlen, Mechanics of fold-and-thrust belts and accretionary wedges. *J. Geophys. Res.* **88**, 1153–1172 (1983).
33. I . Stone, J. E. Vidale, S. Han, E. Roland, Catalog of Offshore Seismicity in Cascadia: Insights Into the Regional Distribution of Microseismicity and its Relation to Subduction Processes. *J. Geophys. Res. Solid Earth* **123**, 641–652 (2018).
34. J. Dieterich, A constitutive law for rate of earthquake production and its application to earthquake clustering. *J. Geophys. Res. Solid Earth* **99**, 2601–2618 (1994).
35. J. Dieterich, V. Cayol, P. Okubo, The use of earthquake rate changes as a stress meter at Kilauea volcano. *Nature* **408**, 457–460 (2000).
36. D . L. Wells, K. J. Coppersmith, New empirical relationships among magnitude, rupture length, rupture width, rupture area, and surface displacement. *Bull. Seismol. Soc. Am.* **84**, 974–1002 (1994).

37. Y. Okada, Surface deformation due to shear and tensile faults in a half-space. *Bullet. Seismol. Soc. Am.* **75**, 1135–1154 (1985).
38. Y. Okada, Internal deformation due to shear and tensile faults in a half-space. *Bullet. Seismol. Soc. Am.* **82**, 1018–1040 (1992).
39. A. R. Nelson, H. M. Kelsey, R. C. Witter, Great earthquakes of variable magnitude at the Cascadia subduction zone. *Quatern. Res.* **65**, 354–365 (2006).
40. V . V. Kostrov, Seismic moment and energy of earthquakes, and seismic flow of rock. *Int. J. Rock Mech. Mining Sci. Geomech. Abst.* **13**, A4 (1976).
41. C . D. Peterson, K. M. Cruikshank, Quaternary Tectonic Deformation, Holocene Paleoseismicity, and Modern Strain in the Unusually–Wide Coupled Zone of the Central Cascadia Margin, Washington and Oregon, USA, and British Columbia, Canada. *JGG* **6**, 1 (2014).
42. T . Ader, J.-P. Avouac, J. Liu-Zeng, H. Lyon-Caen, L. Bollinger, J. Galetzka, J. Genrich, M. Thomas, K. Chanard, S. N. Sapkota, S. Rajaure, P. Shrestha, L. Ding, M. Flouzat, Convergence rate across the Nepal Himalaya and interseismic coupling on the Main Himalayan Thrust: Implications for seismic hazard. *J. Geophys. Res. Solid Earth* **117**, 9071 (2012).
43. S. Rajaure, S. N. Sapkota, L. B. Adhikari, B. Koirala, M. Bhattarai, D. R. Tiwari, U. G. Gautam, P. Shrestha, S. Maske, J. P. Avouac, L. Bollinger, M. R. Pandey, Double difference relocation of local earthquakes in the Nepal Himalaya. *J. Nepal Geol. Soc.* **46**, 133–142 (2013).
44. C . Sippl, B. Schurr, G. Asch, J. Kummerow, Seismicity Structure of the Northern Chile Forearc From >100,000 Double-Difference Relocated Hypocenters. *J. Geophys. Res. Solid Earth* **123**, 4063–4087 (2018).
45. V . Godard, D. L. Bourles, F. Spinabella, D. W. Burbank, B. Bookhagen, G. B. Fisher, A. Moulin, L. Leanni, Dominance of tectonics over climate in Himalayan denudation. *Geology* **42**, 243–246 (2014).
46. L . Dal Zilio, G. Hetenyi, J. Hubbard, L. Bollinger, Building the Himalaya from tectonic to earthquake scales. *Nat. Rev. Earth Environ.* **2**, 251–268 (2021).
47. V . Godard, D. W. Burbank, D. L. Bourles, B. Bookhagen, R. Braucher, G. B. Fisher, Impact of glacial erosion on10Be concentrations in fluvial sediments of the Marsyandi catchment, central Nepal. *J. Geophys. Res.* **117**, 2011JF002230 (2012).
48. J.-P. Avouac, “Mountain building, erosion, and the seismic cycle in the Nepal Himalaya” in *Advances in Geophysics* (Elsevier, 2003), vol. 46, pp. 1–80; https://linkinghub.elsevier.com/retrieve/pii/S0065268703460019.
49. S. Ruiz, R. Madariaga, Historical and recent large megathrust earthquakes in Chile. *Tectonophysics* **733**, 37–56 (2018).
50. A. R. Niemeijer, C. J. Spiers, Compaction creep of quartz-muscovite mixtures at 500°C: Preliminary results on the influence of muscovite on pressure solution. *Geol. Soc. Lond. Spec. Publ.* **200**, 61–71 (2002).
51. N . Brantut, P. Baud, M. J. Heap, P. G. Meredith, Micromechanics of brittle creep in rocks. *J. Geophys. Res. Solid Earth* **117**, 9299 (2012).
52. K. A. McKenzie, K. P. Furlong, M. W. Herman, Regional and Local Patterns of Upper-Plate Deformation in Cascadia: The Importance of the Down-Dip Extent of Locking Relative to Upper-Plate Strength Contrasts. *Tectonics* **41**, e2021TC 007062 (2022).
53. S. Ma, A self-consistent mechanism for slow dynamic deformation and tsunami generation for earthquakes in the shallow subduction zone. *Geophys. Res. Lett.* **39**, 1854 (2012).

54. A. Baker, R. W. Allmendinger, L. A. Owen, J. A. Rech, Permanent deformation caused by subduction earthquakes in northern Chile. *Nat. Geosci.* **6**, 492–496 (2013).
55. Y. Hu, K. Wang, Coseismic strengthening of the shallow portion of the subduction fault and its effects on wedge taper. *J. Geophys. Res. Solid Earth* **113**, 5724 (2008).
56. M. Y. Thomas, H. S. Bhat, Dynamic evolution of off-fault medium during an earthquake: a micromechanics based model. *Geophys. J. Int.* **214**, 1267–1280 (2018).
57. L .-F. Yue, J. Suppe, J.-H. Hung, Structural geology of a classic thrust belt earthquake: the1999 Chi-Chi earthquake Taiwan (Mw=7.6). *J. Struct. Geol.* **27**, 2058–2083 (2005).
58. R. Mallick, R. Burgmann, K. Johnson, J. Hubbard, A Unified Framework for Earthquake Sequences and the Growth of Geological Structure in Fold-Thrust Belts. *Earth* **126**, e2021JB022045 (2021).
59. K. Tsukahara, Y. Takada, Aseismic fold growth in southwestern Taiwan detected by InSAR and GNSS. *Earth Planets Space* **70**, 52 (2018).
60. N . Cubas, P. Agard, R. Tissandier, Earthquake ruptures and topography of the Chilean margin controlled by plate interface deformation. *JGR Solid Earth* **13**, 779–792 (2022).
61. L . Dal Zilio, J. Ruh, J.-P. Avouac, Structural Evolution of Orogenic Wedges: Interplay Between Erosion and Weak Decollements. *Tectonics* **39**, e2020TC 006210 (2020).
62. A. Noda, Forearc basins: Types, geometries, and relationships to subduction zone dynamics. *GSA Bullet.* **128**, 879–895 (2016).
63. E . Tam, Y. Yokoyama, A review of MIS 5e sea-level proxies around Japan. *Earth Syst. Sci. Data* **13**, 1477–1497 (2021).
64. Y. Niwa, T. Sugai, Y. Matsushima, S. Toda, Millennial-scale crustal movements inferred from Holocene sedimentary succession of the Omoto plain, northern Sanriku coast, Northeast Japan: Relevance for modeling megathrust earthquake cycles. *Quat. Int.* **519**, 10–24 (2019).
65. Y. Hu, R. Burgmann, N. Uchida, P. Banerjee, J. T. Freymueller, Stress-driven relaxation of heterogeneous upper mantle and time-dependent afterslip following the 2011 Tohoku earthquake. *J. Geophys. Res. Solid Earth* **121**, 385–411 (2016).
66. A. Helmstetter, B. E. Shaw, Relation between stress heterogeneity and aftershock rate in the rate-and-state model. *J. Geophys. Res. Solid Earth* **111**, 4077 (2006).
67. K. A. Kroll, K. B. Richards-Dinger, J. H. Dieterich, E. S. Cochran, Delayed Seismicity Rate Changes Controlled by Static Stress Transfer. *J. Geophys. Res. Solid Earth* **122**, 7951–7965 (2017).
68. S. Toda, R. S. Stein, T. Sagiya, Evidence from the AD 2000 Izu islands earthquake swarm that stressing rate governs seismicity. *Nature* **419**, 58–61 (2002).
69. S. Toda, R. S. Stein, Central shutdown and surrounding activation of aftershocks from megathrust earthquake stress transfer. *Nat. Geosci.* **15**, 494–500 (2022).
70. E . S. Cochran, J. E. Vidale, S. Tanaka, Earth Tides Can Trigger Shallow Thrust Fault Earthquakes. *Science* **306**, 1164–1166 (2004).
71. C . H. Scholz, Y. J. Tan, F. Albino, The mechanism of tidal triggering of earthquakes at mid-ocean ridges. *Nat. Commun.* **10**, 2526 (2019).
72. T . Parsons, S. Toda, R. S. Stein, A. Barka, J. H. Dieterich, Heightened Odds of Large Earthquakes Near Istanbul: An Interaction-Based Probability Calculation. *Science* **288**, 661–665 (2000).
73. R. S. Stein, A. A. Barka, J. H. Dieterich, Progressive failure on the North Anatolian fault since 1939 by earthquake stress triggering. *Geophys. J. Int.* **128**, 594–604 (1997).
74. F. Cappa, M. M. Scuderi, C. Collettini, Y. Guglielmi, J.-P. Avouac, Stabilization of fault slip by fluid injection in the laboratory and in situ. *Sci. Adv.* **5**, eaau4065 (2019).

75. B. H. Hager, J. Dieterich, C. Frohlich, R. Juanes, S. Mantica, J. H. Shaw, F. Bottazzi, F. Caresani, D. Castineira, A. Cominelli, M. Meda, L. Osculati, S. Petroselli, A. Plesch, A process-based approach to understanding and managing triggered seismicity. *Nature* **595**, 684–689 (2021).
76. E . R. Heimisson, J. D. Smith, J.-P. Avouac, S. J. Bourne, Coulomb threshold rate-and- state model for fault reactivation: application to induced seismicity at Groningen. *Geophys. J. Int.* **228**, 2061–2072 (2022).
77. E . R. Heimisson, P. Segall, Constitutive Law for Earthquake Production Based on Rate-and-State Friction: Dieterich 1994 Revisited. *J. Geophys. Res. Solid Earth* **123**, 4141–4156 (2018).
78. P. Segall, S. Lu, Injection-induced seismicity: Poroelastic and earthquake nucleation effects. *J. Geophys. Res. Solid Earth* **120**, 5082–5103 (2015).
79. R. Almeida, E. O. Lindsey, K. Bradley, J. Hubbard, R. Mallick, E. M. Hill, Can the Updip Limit of Frictional Locking on Megathrusts Be Detected Geodetically? Quantifying the Effect of Stress Shadows on Near-Trench Coupling. *Geophys. Res. Lett.* **45**, 4754–4763 (2018).
80. M. D. Zoback, J. Townend, B. Grollimund, Steady-state failure equilibrium and deformation of intraplate lithosphere. *Int. Geol. Rev.* **44**, 383–401 (2002).
81. B. Gutenberg, C. Richter, *Seismicity of the Earth and Associated Phenomena* (Princeton Univ. Press, ed. 2, 1954).
82. V . L. Stevens, J.-P. Avouac, On the relationship between strain rate and seismicity in the India–Asia collision zone: implications for probabilistic seismic hazard. *Geophys. J. Int.* **226**, 220–245 (2021).
83. D . Schorlemmer, S. Wiemer, M. Wyss, Variations in earthquake-size distribution across different stress regimes. *Nature* **437**, 539–542 (2005).
84. S. Carretier, V. Tolorza, V. Regard, G. Aguilar, M. A. Bermudez, J. Martinod, J.-L. Guyot, G. Herail, R. Riquelme, Review of erosion dynamics along the major N-S climatic gradient in Chile and perspectives. *Geomorphology* **300**, 45–68 (2018).
85. P. Molnar, Climate change, flooding in arid environments, and erosion rates. *Geology* **29**, 1071–1074 (2001).
86. A. T. Codilean, H. Munack, W. M. Saktura, T. J. Cohen, Z. Jacobs, S. Ulm, P. P. Hesse, J. Heyman, K. J. Peters, A. N. Williams, R. B. K. Saktura, X. Rui, K. Chishiro-Dennelly, A. Panta, OCT OPUS database (v.2). *Earth Syst. Sci. Data* **14**, 3695–3713 (2022).
87. C . Placzek, D. E. Granger, A. Matmon, J. Quade, U. Ryb, Geomorphic process rates in the central Atacama Desert, Chile: Insights from cosmogenic nuclides and implications for the onset of hyperaridity. *Am. J. Sci.* **314**, 1462–1512 (2014).
88. D . Lague, The stream power river incision model: evidence, theory and beyond. *Earth Surf. Process. Landf.* **39**, 38–61 (2014).
89. P. Vasquez, F. Sepulveda, A. Quezada, S. Aguilef, C. Franco, N. Blanco, Cartas Guanillos de Norte y Salar de Llamara 1:100.000, regiones de Tarapaca y Antofagasta, Chile (2018).
90. B. Gailleton, S. M. Mudd, Lsdtopotools/lsdtopytools: lsdtopytools (2021).
91. J. Takaku, T. Tadono, K. Tsutsui, Generation of High Resolution Global DSM from ALOS PRISM. *Int. Arch. Photogramm. Remote Sens. Spatial Inform. Sci.* **XL–4**, 243–248 (2014).
92. R. J. Burgette, R. J. Weldon, D. A. Schmidt, Interseismic uplift rates for western Oregon and along-strike variation in locking on the Cascadia subduction zone. *J. Geophys. Res.* **114**, 5679 (2009).
93. Y. Y. Kagan, Seismic moment distribution revisited: I. Statistical results. *Geophys. J. Int.* **148**, 520–541 (2002).
94. R. F. Mereu, A study of the relations between ML, Me, Mw, apparent stress, and fault aspect ratio. *Phys. Earth Planet. In.* **298**, 106278 (2020).

95. V . L. Stevens, S. N. Shrestha, D. K. Maharjan, Probabilistic Seismic Hazard Assessment of Nepal. *Bull. Seismol. Soc. Am.* **108**, 3488–3510 (2018).
96. V . L. Stevens, J.-P. Avouac, Determination of Mmax from Background Seismicity and Moment Conservation. *Bull. Seismol. Soc. Am.* **107**, 2578–2596 (2017).
97. G. Ekstrom, M. Nettles, A. M. Dziewoński, The global CMT project 2004-2010: Centroid-moment tensors for 13,017 earthquakes. *Phys. Earth Planet. In.* **200-201**, 1–9 (2012).
98. A. M. Dziewonski, T. A. Chou, J. H. Woodhouse, Determination of earthquake source parameters from waveform data for studies of global and regional seismicity. *J. Geophys. Res.* **86**, 2825–2852 (1981).
99. G. P. Hayes, G. L. Moore, D. E. Portner, M. Hearne, H. Flamme, M. Furtney, G. M. Smoczyk, Slab2, a comprehensive subduction zone geometry model. *Science* **362**, 58–61 (2018).
